# The impact of the Covid-19 pandemic on the hotel Industry’s economic performance: Evidence from Portugal^☆^

**DOI:** 10.1016/j.heliyon.2023.e15850

**Published:** 2023-05-03

**Authors:** Mário Coutinho dos Santos, José Magano, Jorge Mota

**Affiliations:** a CICEE, Research Center in Economics & Business Sciences, Portugal; b Higher Institute of Business Sciences and Tourism (ISCET), Portugal; c DEGEIT, University of Aveiro, and GOVCOPP, Portugal; d Catholic University of Portugal, Portugal

**Keywords:** Cash-flow at risk, Hotel industry, Covid-19, Monte Carlo simulation

## Abstract

This paper estimates the impact of the Covid-19 pandemic on the economic and financial performance of the Portuguese mainland hotel industry. For that purpose, we implement a novel empirical approach to gauge the impact of the pandemic during the 2020–2021 period in terms of the industry's aggregated operating revenues, net total assets, net total debt, generated cash flow, and financial slack. To that end, we derive and estimate a sustainable growth model to project the 2020 and 2021 ‘Covid-free’ aggregated financial statements of a representative Portuguese mainland hotel industry sample. The impact of the Covid pandemic is measured by the difference between the ‘Covid-free’ financial statements and the historical data drawn from the Orbis and Sabi databases. An MC simulation with bootstrapping indicates that the deviations of the deterministic from the stochastic estimates for major indicators vary between 0.5 and 5.5%. The deterministic operating cash flow estimate lies within plus or minus two standard deviations from the mean interval of the operating cash flow distribution. Based on this distribution, we estimate the downside risk, measured by cash flow at risk, at 1294 million euros. Overall findings shed some light on the economic and financial repercussions of extreme events such as the Covid-19 pandemic, providing us with a better understanding of how to design public policies and business strategies to recover from such an impact.

## 1 Introduction

The outbreak of the Covid-19 pandemic (hereafter, referred to as the ‘pandemic’) triggered unprecedented global disruptions in tourism and hospitality ecosystems and these sectors plunged into a severe economic and financial crisis. Unsurprisingly, such economic, financial and social impacts have a greater negative impact on economies like that of Portugal, which are much more dependent on the performance of the tourism sector than other countries.^1^

At the global hotel industry level, most have been forced to downsize their operations, resulting in significant economic imbalances. Ultimately, some of them may have been driven into financial distress or even insolvency. For example, the 11.1% operating revenue average annual growth rate of the Portuguese mainland hotel industry during the 2014–2019 period was abruptly interrupted by pandemic lockdown measures, travel bans, and other restrictions, triggering unprecedented disruptions and plunging the sector into economic turmoil, with grievous social-economic externalities [1]. Notably, it led to a generalized scale-down of hotel operations, plummeted revenues, fueled workforce layoff, and exposed the ecosystem to economic and financial disarray [5,6]. Although prospects for the recovery of the tourism sector remained relatively unsteady, governmental policymakers and hotel ecosystem participants alike strove to map out alternative feasible recovery paths (and pace) for designing post-pandemic resilient and sustainable recovery strategies for the industry (e.g., Ref. [7]).

The most recent research on the topic examines either public policy aimed at mitigating the impact of COVID-19 on tourism (e.g., Refs. [[8], [9], [10]]), or explores particular performance dimensions of specific segments of the hotel industry, such as listed hotels (e.g., Refs. [[11], [12], [13], [14]]).^2^ Yet, the development of blueprints to support the design of prospective strategic scenarios requires the availability of a comprehensive and quantitative assessment of the magnitude of the economic, financial, and social impacts of the Covid-19 shock. To our knowledge, no such assessment has been carried out, motivating us to perform this study.

The main research objective of this paper is to quantitatively assess the potential economic and financial shocks on the performance of the Portuguese hotel industry. Data are based on approximately 1000 hotels in mainland Portugal in the period 2020–2021.

Besides providing evidence of the aggregated pandemic's impact on the economic and financial condition of the industry, this work also contributes to the literature by applying a novel methodological approach. First, it derivates and estimates a deterministic business model for the sector, anchored on the maximum sustainable growth rate (SGM) conceptual framework to gauge the impact of the pandemic during the 2020–2021 period in terms of operating revenues, net total assets, net total debt, operating cash flow, and financial slack.^3^ Next, this approach combines with a robustness check that incorporates stochastic variability into the deterministic base case by applying Monte Carlo (MC) methods with bootstrapping to measure the downside risk of our hotel industry sample using the cash-flow-at-risk (*CFaR*) conceptual framework.

## 2 Conceptual background

### 2.1 Maximum sustainable growth framework

Tourism and hospitality industries are exposed to a wide spectrum of risks. Therefore, enterprise risk management (ERM) — risk identification, measurement, and management — is instrumental in helping firms to manage their value creation objectives, particularly in terms of mitigating financial distress and optimizing risk portfolio (e.g., Refs. [[25], [26], [27], [28], [29]]).^4^

At the hotel firm level, the measurement of the expected impact of downside risk factors on value creation should be a primary managerial concern (e.g., Refs. [27,[33], [34], [35]]. Further, there is abundant and compelling evidence that the volatility in corporate accounting aggregates, such as net income and operating cash flows, is related to value creation (e.g., Smithson & Simkins, 2005 [33]). Under well-diversified firm ownership, risk management can be expected to be positively related to a firm's value, which could limit the expected costs of financial distress, manage financial slack, reduce tax liability, and mitigate suboptimal resource allocation (e.g., Ref. [34]).

To estimate the economic and financial impact of the Covid-19 pandemic on the Portuguese mainland hotel industry in 2020 and 2021, we develop a novel empirical, methodological approach based on the SGM framework. SGM builds on the percentage-of-sales method's standard assumptions that the stocks of the balance sheet accounts are optimized in relation to the current level of sales and vary in proportion to sales; and that depreciation and amortization are not an available source of funds because it is assumed that the same amount is applied in restoring fixed assets operational functionality (e.g., Refs. [[36], [37], [38]]).^5^

Under the maximum annual percentage increase in operating revenue — *g* — a firm can sustain, keeping constant at the pre-pandemic levels (2019): (*i*) fixed assets utilization, proxied by the net fixed assets-to-operating revenue ratio; (*ii*) after-tax operating revenue profitability, measured by the net income-to-operating revenue ratio; (*iii*) capital structure gauged by the debt-to-equity ratio; and (*iv*) the retention rate of earnings, measured by the complement of the dividend payout ratio, and without resorting to incremental external funding.

Financial slack (FS) is a readily available liquidity cushion in the form of excess cash holdings and debt capacity, which provides financing flexibility by mitigating the impact of adverse liquidity shocks, and financial distress and by moderating suboptimal allocative behavior, namely in the form of underinvestment (e.g., Ref. [12]).^6^ Therefore, for precautionary reasons, firms tend to accumulate liquid assets, such as cash and equivalents, as an ‘insurance’ against liquidity shortfalls arising in adverse states of cash flow generation and to avoid asset fire sales, raising externally costly unanticipated funding, or incurring inefficient underinvestment (e.g., Refs. [[39], [40], [41], [42]]).

Under this framework, firms with higher asset systematic riskiness and costlier access to external capital markets tend to carry larger cash holdings on their balance sheets. We measure excess cash holdings as the difference between “Cash & Equivalents” and the “Liquidity Buffer” balances (e.g., Refs. [[43], [44], [45], [46]]).^7^ Conceptually, debt capacity is the incremental borrowing required to sustain the capital market's perception of a firm's current aggregate asset systematic riskiness. Or restated, the maximum amount that could optimally be borrowed at the current risk-adjusted marginal cost of debt (e.g., Refs. [39,47,48]).

### 2.2 Cash-flow-at-risk

Another valuable tool to assess non-financial firms' downside risk is cash flow at risk (*CFaR*). *CFaR* is a composite measure of the maximum decrease in expected cash flows associated with the uncertainty of risk factors, given a pre-defined confidence level, for a given period, which Stein et al. [49] define “as the probability distribution of a company's operating cashflows over some horizon in the future, based on information available today”. Taking a prespecified timeframe and statistical confidence level, the *CFaR* approach to downside risk measurement estimates the maximum shortfall of cash a firm is willing to accept and, therefore, its overall liquidity risk over a given period (e.g., Refs. [27,[49], [50], [51]].^8^, Moreover, since all risk exposures can be aggregated into a single metric, *CFaR* provides quantitative information, at least accurate on average, helping to guide managerial decision-making (e.g., Refs. [34,53,54]). Indeed, ‘it is the “lower tail” of the cash flow distribution that can have costly consequences, such as insufficient funds to carry out the company's investment program or even bankruptcy’ [54]; *CFaR* provides a measure of such lower tail effects, which we estimate by bootstrapping the Portuguese mainland hotel industry model for robustness-checking purposes, following Alexander [55].

It should be emphasized that the data panel used in the deterministic methodological approach features an inherent statistical significance limitation, which inhibits inferential testing. The estimation of *CFaR* with Monte Carlo (MC) simulation has been addressed in the literature, namely, through examples that suggest that this numerical tool is effective for solving problems in finance that involve closed-form analytical solutions that are too complex or impossible to determine (e.g., Refs. [56,57]). Besides the benefit of efficiently dealing with complexity, another advantage of MC is its inherent randomness, which is essential for simulating real-life random systems [58]. This method is, therefore, an obvious choice for tackling the *CFar* estimation as a robust check in our study's approach. Hence, we use MC bootstrapping computational methods to perform the number of trials necessary in each simulation experiment to generate a numerical approximation to the true distribution of the output variable at the standard 95% confidence level.^9^

## 3 Methods

### 3.1 Research design

This paper examines the impact of the Covid-19 pandemic period during the 2020 and 2021 sampling periods on the Portuguese mainland hotel industry. Specifically, we estimate the pandemic effect in terms of total net assets, total net debt, operating cash flow, and financial flexibility, for a representative sample of Portugal's mainland hotels (hereafter, referred to as the ‘sample').

The empirical implementation strategy is designed in five steps. First, we estimate aggregate balance sheets, income, and operating cash flow statements for the 2014–2021 sampling period, drawing data from the Orbis/Sabi databases. Second, we derive a steady-state version of SGM and estimate the operating revenue sustainable growth rate to forecast the sample's financial statements for 2020 and 2021.^10^ Regarding the third step, we project 2020 and 2021 aggregate balance sheets, income statements and cash flow statements, which are unconditioned by the occurrence of the Covid-19 pandemic. Fourth, we measure the (deterministic) impact of the Covid-19 pandemic as the difference between the projected and observed 2020's and 2021's aggregated operating revenues, net total assets, total net debt, operating cash flow, and financial slack. Fifth, we run a Monte Carlo simulation experiment to check for the robustness of the deterministic 2019 base case in terms of the output variables required to compute the *CFaR*.

### 3.2 Data

Economic, financial, and operating data for this research were drawn from INE (Statistics Portugal), Sabi, and Orbis, covering the 2010–2021 sampling period. However, to minimize the spillovers of the financial assistance program signed between Portugal and the International Monetary Fund, the European Union, and the European Central Bank, encompassing the application of a three-year economic adjustment program (2011-mid-2014), we restricted the sampling period to 2014–2019.

Results of summary statistics and parametric tests for equality of means document that the variables used in the deterministic model do not exhibit, at the standard confidence levels, statistically significant differences in means between the sampling subperiods of 2010–2019 and 2014–2019. These results support the consistency of using the 2014–2019 subsampling period for this empirical analysis and segmenting hotels by star category.^11^

To be included in the sample, a hotel must comply with the following criteria: (*i*) be included in the National Register of Tourism Enterprises (RNET) database, with an assigned fiscal number; (*ii*) be established and operating on Portugal's mainland (*iii*) be a star classified hotel or hotel-apartment; (*iv*) be active for the entire sampling period.^12^ The population of hotels and hotel-apartments was drawn from the INE database for the 2019–2021 period (Table 1).

**Table 1:** | Hotel population and sample. The table reports the population of hotels and hotel-apartments from 2019 to 2021 (a), and the sample's distribution in 2019 (b). Hotel enterprises are legal, fiscal established entities. Each hotel enterprise may own more than one hotel and hotel-apartment unit.

Portugal mainland industry^a^		Total	Percent
Hotels	2019	1286	91.3
	2020	1098	91.7
	2021	1260	91.8
Hotel-Apartments	2019	122	8.7
	2020	100	8.3
	2021	113	8.2
Total	2019	1408	100.0
	2020	1198	100.0
	2021	1373	100.0
Sample (hotels and hotel-apartments)^b^			
Hotel enterprises		972	
Hotel and hotel-apartment units		1057	

A search in the RNET database yielded a sample of 1282 hotel units with assigned fiscal numbers and star classifications that met the above-mentioned criteria. Finally, we identified 972 entities in Orbis/Sabi databases that matched that set and reported complete data for the 2019–2021 period, which corresponded to 1057 hotel and hotel-apartment units. For this sample, we extracted economic, financial, and operating data at the hotel business firm level (Table 1).^13^

Data on income statement items, such as labor costs, other operating costs, financial revenues, and financial expenses, were drawn from the Sabi database. In addition, data on the interest coverage ratios and the debt spreads associated with the synthetic credit ratings were collected from Prof. Aswath Damodaran's website^14^; 10-year government bond yields for Portugal and triple-A rating countries were collected from the European Central Bank's - Statistical Data Warehouse.

### 3.3 SGM modeling and estimation

Aggregate balance sheets, income, and cash flow statements were estimated using the variable specified in Appendix II. Financial slack is modeled as the sum of excess cash holdings (ECH) and debt capacity (DC). ECH is measured as the difference between the book value of cash and equivalents, and the minimum cash balance requirements (e.g., Ref. [44]).^15^ We use the defensive interval ratio (DIR) to estimate the short-term liquidity needs in terms of the number of days a hotel could operate resorting only to its current assets (e.g., Ref. [60]):

(1) $$DIR=\frac{{Currednt\;Assests}_{t}}{{Daily\;Operating\;Expenses}_{t}}$$

where current assets include cash, marketable securities, and net receivables; and daily operational expenses are measured by the sum of the cost of sales, operating costs, and net interest rate divided by 360.

We define DC as the maximum amount of borrowing lenders are willing to extend to an investment-grade rated firm based on its interest coverage ratio and the debt spread associated with its rating notation (see, e.g., Ref. [61]). Hotel's DC is specified as:

(2) $$Debt\;Capacity=\frac{\frac{Earnings\;Before\;Interests_{t}\;\&\;Taxes_{t}}{Interest\;Coverage\;Ratio_{t}}}{r_{F}+spread}$$

where *r*_*F*_ denotes the rate of return on a riskless asset; and spread, the debt's default risk premium.

We estimate hotel synthetic rating notations, interest coverage ratios, and the debt default spreads associated with them, using the model by Damodaran [62], parametrized according to Aswath Damodaran's website (see Table 2). The model uses the operating income (EBIT) and the net interest expense as inputs to estimate the interest coverage ratio, which is extensively used by Standard and Poor’s and Moody’s, two leading international rating agencies.

**Table 2:** | Interest coverage ratios, synthetic credit ratings, and debt spreads. This table presents data on the interest coverage ratios and the debt spreads associated with the synthetic credit ratings collected from Prof. Aswath Damodaran's website (http://pages.stern.nyu.edu/∼adamodar/New_Home_Page/dataarchived.html, accessed on 9 May 2022). The table also reports the sample's average interest coverage ratio, synthetic credit ratings, and debt spreads for the: Base Case (see [1]); the interest coverage ratios, synthetic credit ratings, and debt spreads are estimated under the assumption of Covid absence, and stability relating with the base case (see [2] and [4]); and the real aggregate values for the years 2020_r_ and 2021_r_, respectively (see [3] and [5]).

Damodaran's interest coverage ratios, synthetic credit ratings and debt spreads							
Interest coverage ratio		Synthetic Rating	2019 spread (percent)		2020 spread (percent)		2021 spread (percent)
−100000	0.199	D2/D	19.4		15.1		17.4
0.2	0.650	C2/C	14.5		11.0		13.1
0.65	0.800	Ca2/CC	11.1		8.6		10.0
0.8	1.250	Caa/CCC	9.0		8.2		9.5
1.25	1.500	B3/B-	6.6		5.2		6.0
1.5	1.750	B2/B	5.4		4.2		4.9
1.75	2.000	B1/B+	4.5		3.5		4.0
2	2.250	Ba2/BB	3.6		2.4		2.8
2.25	2.500	Ba1/BB+	2.5		2.0		2.3
2.5	3.000	Baa2/BBB	2.0		1.6		1.7
3	4.250	A3/A-	1.6		1.2		1.3
4.25	5.500	A2/A	1.4		1.1		1.2
5.5	6.500	A1/A+	1.3		1.0		1.1
6.5	8.500	Aa2/AA	1.0		0.8		0.9
8.5	10,000,000	Aaa/AAA	0.8		0.7		0.7
			[1] Base Case	[2] projection(p)<	[3] real(r)<	[4] projection(p)<	[5] real(r)<
Average interest coverage ratio of the sample			7.6930	7.6930	−8.8915	7.6930	0.2795
Synthetic credit rating of the sample			Aa2/AA	Aa2/AA	D2/D	Aa2/AA	C2/C
Debt spread (percent) of the sample			1.0	0.8	15.1	0.9	13.1

### 3.4 MC approach

**Fig. 1:**
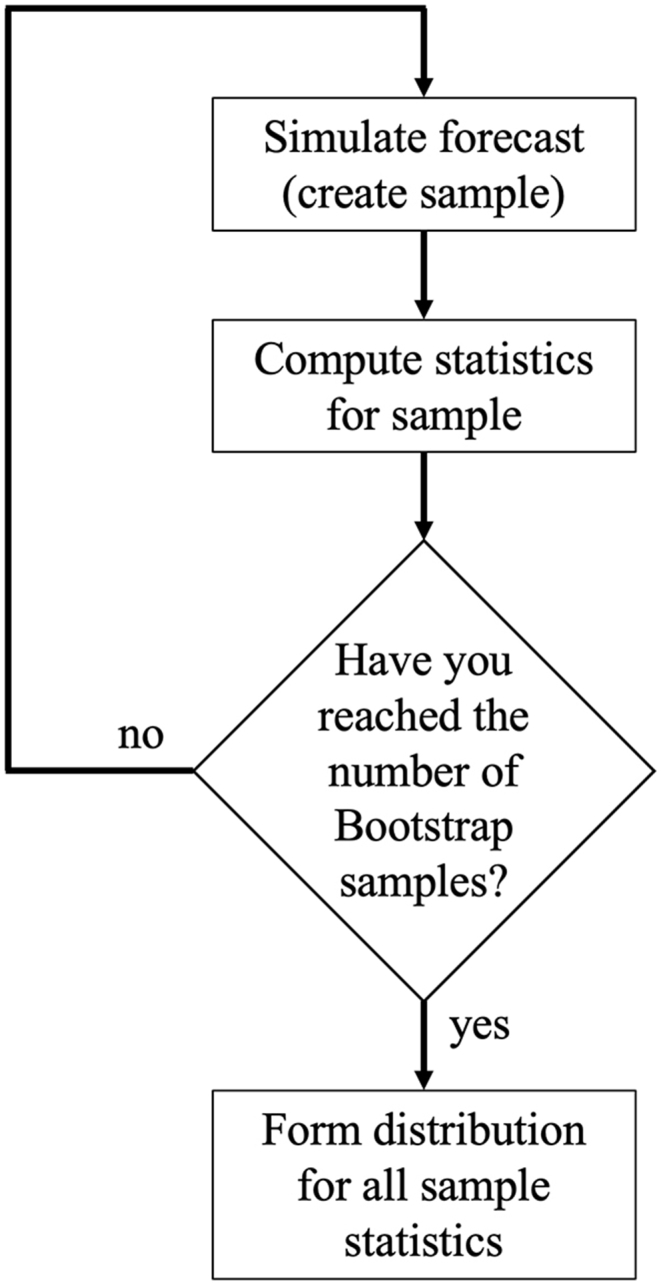
Bootstrap multiple-simulation method. Source: Crystal Ball User Guide.

We use MC computational numerical methods with bootstrapping to produce forecasts of the 2020 and 2021 aggregated operating revenues, net total assets, net total debt, operating cash flow, financial slack, debt capacity, and excess cash holdings to check for the robustness of the deterministic model. A single independent variable — operating revenues sustainable growth rate — is randomized, specified as a triangular distribution, and heuristically calibrated as follows: minimum = 0%; likeliest value = 12%; and maximum = 17%.^16^ The MC simulation follows a bootstrap multiple-simulation approach, repeatedly simulating the model and then creating a distribution of the statistics from each simulation, as depicted in Fig. 1.

## 4 Results

### 4.1 SGM estimation

Data in Table 1 document that during the Covid-19 shock, the hotel population diminished relatively to 2019. However, it only partially recovered in 2021 without achieving pre-pandemic levels. We estimate the 2019 ‘Covid-19-free’ operating revenue sustainable growth rate, using the steady-state SGM model derived in Appendix I, at 12.0% (see Table 3). We used this growth rate to project pro forma aggregate balance sheets, income, and cash flow statements for the 2020 and 2021 periods (see Appendix II for the specification of the variables).

**Table 3:** | Operating revenue sustainable growth rate estimation. The table reports the estimates of the operating revenue sustainable growth rate using a steady-state version SGM (see Appendix I) under the following assumptions: the values of the variables used were taken directly from the databases, without intermediate estimations; Other current liabilities_2019_ = Current liabilities_2019_ – Payables_2019_; Retention rate_2019_ = 1- Dividend paid out_2019_/Net income_2019_. The specification of all variables can be accessed at: https://help.bvdinfo.com/mergedProjects/64_en/Home.htm.

Operating revenue	*S*	2019
Cash & equivalents/Operating revenue	*C*&*E*/*S*	0.17
Receivables/Operating revenue	*RCV*/*S*	0.05
Inventory/Operating revenue	*INV*/*S*	0.06
Other current assets/Operating revenue	*OCA*/*S*	0.44
Fixed assets/Operating revenue	*FAS*/*S*	2.26
Payables/Operating revenue	*PAY*/*S*	0.08
Other current liabilities/Operating revenue	*OCL*/*S*	0.37
Net income/Operating revenue	*NIC*/*S*	0.12
Retention rate	*r*	1.55
Debt/Equity	*D*/*E*	0.50
	*g*	12.0%

Panels A, B, and C of Table 4 present the estimation of the sample's aggregate financial statements for the 2020 and 2021 periods, based on the 2019 period's SGR estimate unconditioned by the Covid-19 outbreak. The table presents the 2019 base case (column [1]); the deterministic estimations for 2020 and 2021 aggregate pro forma balance sheet, income, and cash flow statements (columns [2,3]); the 2020 and 2021 real aggregate balance sheet, income, and cash flow statements (columns [4,5]); and the estimates of the impact of the Covid-19 pandemic on the output variables measured by the differences between the 2020–2020 and 2021–2021 real aggregate values and the deterministic estimates (columns [5,6]).

**Table 4:** | Pandemic impacts on income statement, balance sheet, and cash flow statement variables (2020–2021) (unit: 10^3^ euros). The table reports the estimates on the economic and financial impact (income statement, balance sheet, and cash flow statement variables) that the pandemic had on the Portuguese hotel sector, presenting the Base Case as the year 2019 (see [1]), the deterministic estimates for the period 2020 + 2021 (see [2]), the real aggregate values for the same period (see [3]), the impact of the Covid-19 pandemic on the output variables measured by the difference between the 2020–2021 real values and the aggregate deterministic estimates (see [4]), and under the following assumptions: the values of the variables used were taken directly from the databases, without intermediate estimations; Net interest expense_*t*_ = (Net interest expense_t-1_/Non-current liabilities_t-1_ * Funding needs_*t*_) + Net interest expense_*t-1*_; Funding needs_*t*_ = Total assets_*t*_ – Equity_*t*_ – Non-current liabilities_*t*_ – Current liabilities_*t*_; Paid out dividend_*t*_ = Net income_*t*_ - Δ (Equity_*t*_ – Equity_*t-1*_) under the assumption that the issuance and repurchase of shares are equivalent to each other. The specification of all variables can be accessed at: https://help.bvdinfo.com/mergedProjects/64_en/Home.htm.

Panel A | Income statement				
	[1] Base Case	[2] 2020_p_+2021_p_	[3] 2021_r_+2021_r_	[4] (2020_r_+2021_r_)-(2020_p_+2021_p_)<
Operating revenue	3,492,010	8,288,356	2,970,967	−5,317,389
Cost of sales	389,307	924,027	331,730	−592,297
Operating costs	2,222,072	5,274,133	2,570,761	−2,703,372
Depreciation	311,560	739,494	570,707	−168,787
EBIT	569,071	1,350,702	−502,231	−1,852,933
Net interest expense	73,972	243,408	118,358	−125,051
P/L before tax	495,099	1,107,294	−620,588	−1,727,882
Income taxes	88,017	196,851	−42,799	−239,650
Net income	407,082	910,443	−547,332	−1,457,775
Paid out dividends	−224,260	−501,561	522,013	1,023,574
Panel B | Balance sheet				
	[1] Base Case	[2] 2020_p_+2021_p_	[3] 2021_r_+2021_r_	[4] (2020_r_+2021_r_)-(2020_p_+2021_p_)<
Fixed assets	7,898,010	18,746,087	13,941,042	−4,805,045
Current assets	1,914,164	4,543,307	2,189,331	−2,353,976
TOTAL ASSETS	9,812,174	24,723,395	17,098,106	−7,625,289
Equity	8,693,769	11,445,230	7,622,391	−3,822,839
Liabilities	5,139,679	13,278,165	9,475,715	−3,802,450
Non-current liabilities	3,554,090	10,003,635	6,980,009	−3,023,625
Current liabilities	1,585,589	3,274,531	2,495,706	−778,825
TOTAL EQUITY + LIABILITIES	9,812,174	24,723,395	17,098,106	−7,625,289
Panel C | Cash flow statement				
	[1] Base Case	[2] 2020_p_+2021_p_	[3] 2021_r_+2021_r_	[4] (2020_r_+2021_r_)-(2020_p_+2021_p_)<
Operating Cash Flow	718,642	1,649,937	23,375	−1,626,562
Δ Working capital	620,421	332,467	−555,801	−888,268
Net Operating Cash Flow	98,221	1,317,470	579,176	−738,294

The deterministically estimated aggregate impacts inflicted on our Portuguese mainland hotel industry sample over the 2020–2021 pandemic period are presented in Table 5:

**Table 5:** | Pandemic impacts (2020–2021). This table presents: the Base Case as the year 2019 (see [1]); the real aggregate values for the period 2020 + 2021 (see [2]); the deterministic estimates for the same period (see [3]); the impact of the Covid-19 pandemic on the output variables measured by the difference between the 2020–2021 the real values and aggregate deterministic estimates (see [4]); and the percent of estimates, calculated as [4]/[3].

Output variables	[1] Base case (10^3^ euros)<	[2] 2021 + 2021_r_ (10^3^ euros)	[3] 2020 + 2021_p_ (10^3^ euros)	[4] Real – Estimate (10^3^ euros)<	[5] Percent of estimates
Operating revenues	3,492,010	2,970,967	8,288,356	−5,317,389	−64.2
Net Total Assets	9,812,174	17,098,106	24,723,395	−7,625,289	−30.8
Net Total Debt	2,949,924	6,012,276	8,569,634	−2,557,358	−29.8
Net Income	407,082	−547,332	910,443	−1,457,775	−160.1
Operating Cash Flow	718,642	579,176	1,317,470	−738,294	−56.0
Financial Slack	−127,236	1,529,487	8,078,267	- 6,548,780	−81.1
Debt capacity	3,308,615	8,357,450	17,801,258	- 9,443,808	−53.1
Excess cash holdings	118,239	152,047	280, 644	−128,597	−45.8

The estimated economic impacts measured by the aggregated operating revenues, net income, and operating cash flow are: a 64.2% reduction (−5317 million euros); a 160.1% decrease (−1457 million euros); and a 56.0% drop (−738 million euros), respectively. The financial repercussions, gauged by the variation in the non-current liabilities and the financial slack, are: a 29.9% increase (+2557 million euros); and an 87.1% decline (−9316 million euros), respectively. The latter impact is the compound effect of the 53.1% fall in debt capacity (−9444 million euros) and the 45.8% reduction in the excess cash holdings (−129 million euros).

Despite the reported aggregate impacts of 2020 and 2021, we must recognize that, after a severe decrease in hotel demand in 2020, some recovery was seen in 2021. Accordingly, our model presents different impact variations between the estimated and real outcomes from 2020 to 2021, of which it is worth mentioning the following (*i*) a 50 million euro increase in operating revenues (1.9%); (*ii*) a 1238 million euro drop in the industry's net total assets (69.4%); (*iii*) an 894 million euro increase in the sector's non-current liabilities balance (107.4%); (*iv*) a 440 million euro increase in net income (46.4%); (*v*) a 369 million euro increase in the operating cash flow (44.4%); (*vi*) a 4144 million euro drop in financial slack (344.5%); (*vii*) a 4998 million euro drop in financial slack (224.9%); and (*viii*) a 4 million euro drop in financial slack (6.6%).

**Fig. 2:**
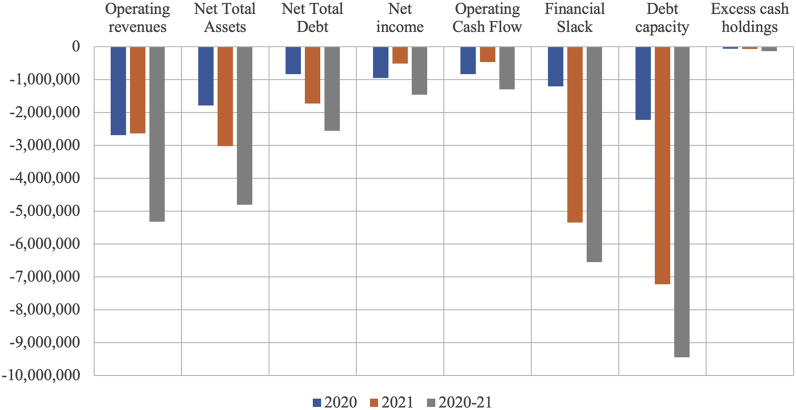
The impact of the Covid-19 pandemic: 2020, 2021, and 2020–21 (unit: 10^3^ euros).

To summarize, in 2021, net income and operating cash flow improved due to growing demand, whereas net total assets, net total debt, and financial slack worsened (Fig. 2).

The launch of the vaccination campaign by the end of 2020 may have, to a certain extent, lessened tourist travel restrictions, creating the conditions necessary for some recovery in hotel occupancy rates in 2021 and, consequently, in operating revenues. However, as shown in Fig. 2, the difference between 2020 and 2021 operating revenues is relatively marginal. We conjecture that this might have been the effect of the phasing out in 2021 of the governmental Covid-19 mitigating measures in place.

Debt capacity and excess cash holdings are the two sources of financial flexibility required to mitigate potential liquidity shortfalls and suboptimal allocative behavior in adverse states of the world, such as during the Covid-19 outbreak (See Panel D in Appendix II for the specification of the variables). Results document that over the 2020–2021 period, the hotel industry suffered a major fall-off in financial flexibility due, first and foremost, to debt capacity contraction, which seriously curtails potential recovery funding needs, notably in the new context of central banking's monetary tightening policy. The breakdown of financial slack indicates that borrowing capacity is its main determinant (see Table 5 and Fig. 2).

To sharpen the analysis of the results, we estimate the 2020 and 2021 impacts of the output variables at the hotel unit level (see Table 6). As expected, the impacts on accounting economic aggregates, such as operating revenues, net income, and operating cash flow, were more negative in the first year of the pandemic.

**Table 6:** | Impacts of the pandemic per hotel unit in 2020 and 2021. This table presents: the impact per hotel unit of the Covid-19 pandemic on the output variables, calculated as the difference between the real value and the deterministic estimate in 2020 (see [1]), and 2021 (see [2]); and the variation of those impacts in 10^3^ euros (see [3]) and percentage (see [4]). The number of hotel units of the sample in 2020 and 2021, respectively, 899 and 1,030, were estimated from the real sample size in 2019 (1057 hotel units) in proportion to the real total hotel units in Portugal mainland reported by INE (1408 in 2019, 1198 in 2020, and 1373 in 2021). (INE, 2021).

Output variables	[1] 2020 Impact per hotel unit: [Real – deterministic estimates]/No. hotel units in 2020 (10^3^ euros)	[2] 2021 Impact per hotel unit: [Real – deterministic estimates]/No. hotel units in 2021 (10^3^ euros)	[3] Variation per hotel unit from 2020 to 2021 (10^3^ euros): [2]-[1]	[4] Percent variation per hotel unit from 2020 to 2021
Operating revenues	−2985	−2557	429	−14.4
Net Total Assets	−1984	−2933	−949	47.8
Net Total Debt	−925	−1675	−750	81.1
Net Income	−1055	−494	561	−53.2
Operating Cash Flow	−925	−449	476	−51.5
Financial Slack	−1338	−5190	−3853	288.0
Debt capacity	−2472	−7011	−4538	183.6
Excess cash holdings	−69	−64	5	−7.0

### 4.2 Robustness check: Monte Carlo simulation

As previously described, we followed a bootstrap MC multiple-simulation approach, repeatedly running the model. As such, ten simulation experiments were performed, each one with the number of trials required to generate a numerical approximation to the distribution of the output variables.^17^ Results are summarized in Table 7.^18^

**Table 7:** | Pandemic impacts – simulation results. In this table, impacts are calculated as the difference between the real values and the Monte Carlos simulation estimates (impacts measured in 10^3^ euros), and the percentage of these estimates (ratio between the impact and the real value). The precision control settings were activated in Crystal Ball and set to ensure the simulation trials would stop when the standard 95% confidence level was reached.

Trials	Simulation experiments										Mean of means
	1	2	3	4	5	6	7	8	9	10	
	50	50	50	50	50	50	50	50	50	50	
Operating revenues											
Mean	8,007,468	8,060,278	7,998,740	8,044,370	8,034,102	8,099,004	7,947,705	8,134,236	8,052,962	8,034,122	8,041,299
Standard deviation	450,331	365,999	376,435	307,269	335,789	379,379	446,442	416,617	381,428	422,401	388,209
Impact (10^3^ euros)	9,090,638	9,037,829	9,099,366	9,053,737	9,064,004	8,999,102	9,150,401	8,963,870	9,045,144	9,063,984	9,056,808
Impact (percentage)	113.5	112.1	113.8	112.6	112.8	111.1	115.1	110.2	112.3	112.8	112.7
Net total assets											
Mean	23,885,532	24,043,058	23,859,498	23,995,606	23,964,978	24,158,574	23,707,264	24,263,668	24,021,237	23,965,038	23,986,445
Standard deviation	1,343,295	1,091,740	1,122,870	916,556	1,001,628	1,131,653	1,331,696	1,242,729	1,137,765	1,259,984	1,157,991
Impact (10^3^ euros)	−6,787,426	−6,944,952	−6,761,392	−6,897,500	−6,866,872	−7,060,468	−6,609,158	−7,165,562	−6,923,131	−6,866,932	−6,888,339
Impact (percentage)	−28.4	−28.9	−28.3	−28.7	−28.7	−29.2	−27.9	−29.5	−28.8	−28.7	−28.7
Net total debt											
Mean	7,863,357	7,996,383	7,841,280	7,956,239	7,930,379	8,094,007	7,712,722	8,182,854	7,977,961	7,930,506	7,948,569
Standard deviation	1,134,950	956,880	948,802	774,494	846,357	956,244	1,125,151	1,050,063	961,319	1,064,609	981,887
Impact (10^3^ euros)	9,234,749	9,101,723	9,256,826	9,141,867	9,167,727	9,004,099	9,385,384	8,915,252	9,120,145	9,167,600	9,149,537
Impact (percentage)	117.4	113.8	118.1	114.9	115.6	111.2	121.7	109.0	114.3	115.6	115.2
Net income											
Mean	879,005	884,727	878,063	883,004	881,892	888,925	872,531	892,742	883,933	881,894	882,672
Standard deviation	48,800	39,658	40,782	33,287	36,378	41,103	48,376	45,147	41,330	45,771	42,063
Impact (10^3^ euros)	−299,829	−305,551	−298,887	−303,828	−302,716	−309,749	−293,355	−313,566	−304,757	−302,718	−303,495
Impact (percentage)	−34.1	−34.5	−34.0	−34.4	−34.3	−34.9	−33.6	−35.1	−34.5	−34.3	−34.4
Operating cash flow											
Mean	1,332,897	1,329,954	1,333,629	1,331,042	1,331,599	1,327,586	1,336,479	1,325,352	1,330,352	1,331,383	1,331,027
Standard deviation	26,720	21,900	22,545	18,460	20,115	22,787	26,482	24,946	22,676	25,188	23,182
Impact (10^3^ euros)	44,543	47,486	43,811	46,399	45,841	49,854	40,961	52,088	47,088	46,057	46,413
Impact (percentage)	3.3	3.6	3.3	3.5	3.4	3.8	3.06%	3.9	3.5	3.5	3.5
Financial slack											
Mean	8,329,951	8,301,003	8,333,433	8,308,932	8,314,553	8,280,469	8,361,543	8,262,287	8,305,212	8,315,326	8,311,271
Standard deviation	240,764	194,632	199,436	162,399	177,805	200,972	238,491	238,491	203,389	225,078	208,146
Impact (10^3^ euros)	−2,317,675	−2,288,727	−2,321,156	−2,296,655	−2,302,277	−2,268,192	−2,349,266	−2,250,011	−2,292,935	−2,303,050	−2,298,994
Impact (percentage)	−27.8	−27.6	−27.9	−27.6	−27.7	−27.4	−28.1	−27.2	−27.6	−27.7	−27.7
Debt capacity											
Mean	17,307,578	17,419,005	17,287,769	17,384,576	17,362,909	17,501,484	17,180,219	17,577,051	17,403,774	17,363,812	17,378,818
Standard deviation	956,880	778,808	801,754	654,855	715,285	808,068	948,816	886,185	811,029	898,332	826,001
Impact (10^3^ euros)	−15,930,138	−16,041,565	−15,910,328	−16,007,136	−15,985,469	−16,124,044	−15,802,779	−16,199,611	−16,026,334	−15,986,372	−16,001,378
Impact (percentage)	−92.0	−92.1	−92.0	−92.1	−92.1	−92.1	−92.0	−92.2	−92.1	−92.1	−92.1
Excess cash holdings											
Mean	271,133	272,921	270,837	272,382	272,035	274,232	269,109	275,425	272,673	272,035	272,278
Standard deviation	15,248	12,393	12,746	10,404	11,370	12,846	15,117	14,107	12,915	14,303	13,145
Impact (10^3^ euros)	−818,465	−820,253	−818,169	−819,714	−819,366	−821,564	−816,441	−822,757	−820,005	−819,367	−819,610
Impact (percentage)	−301.9	−300.6	−302.1	−301.0	−301.2	−299.6	−303.4	−298.7	−300.7	−301.2	−301.0
Cash flow at risk											
*CFaR* (10^3^ euros)	1,289,972	1,294,724	1,296,050	1,299,094	1,299,968	1,293,503	1,291,871	1,289,014	1,294,308	1,288,348	1,293,685

In each experiment, cash flow at risk was estimated as the operating cash flow for which the accumulated probability of occurrence is 5%, or, in other words, the likelihood of exceeding *CFaR* is 95%. The Monte Carlo simulation results are very close to those obtained with the deterministic approach described in section 4. Table 8 exhibits the robustness check estimates performed using Monte Carlo simulation methods with bootstrapping for the net total assets, net total debt, operating cash flow, and financial slack at risk, as well as the aggregate impact of the Covid-19 pandemic for the 2020–21 period. These suggest that, on average, the absolute deviations between deterministic and stochastic estimates at 95% confidence level are relatively minor, oscillating between 0.5 and 5.5%.

**Table 8:** | Robustness checks on the pandemic impacts (2020–2021). This table presents: the real aggregate impact for the 2020–21 period [1]; the deterministic estimations for output variables (see [2]); the Monte Carlo (MC) estimations for output variables (see [3]); the aggregate impact for the 2020–21 period considering MC estimates in 10^3^ euros [4] and percent [5]; the differences between the pandemic deterministic and MC impacts as a percent of real [6]; deviation of the two estimates as a percent of the deterministic estimates [7].

Output variables	[1] Real 2020–21 (10^3^ euros)	[2] Deterministic estimates 2020–21 (10^3^ euros)	[3] MC estimates 2020–21 (10^3^ euros)<	[4] Real-MC estimates 2020–21 (10^3^ euros)	[5] Real-MC est. As percent of MC estimates	[6] percent<	[7] percent<
						[3] – [1]	[4]/[3]
Operating revenues	2,970,967	8,288,356	8,041,299	−5,070,332	−63.1	1.1	−1.7
Net Total Assets	17,098,106	24,723,395	23,986,445	−6,888,339	−28.7	2.1	−6.9
Net Total Debt	6,012,276	8,569,634	7,948,569	−1,936,292	−24.4	5.5	−18.4
Net Income	- 547,332	910,443	882,672	−1,430,003	−162.0	−1.9	1.2
Operating Cash Flow	579,176	1,317,470	1,331 027	−751,851	−56.5	−0.5	0.8
Financial Slack	1,529,487	8,078,267	8,180,446	−6,650,959	−81.3	−0.2	0.3
Debt capacity	8,357,450	17,801,258	17,378,818	−9,021,368	−51.9	1.1	−2.2
Excess cash holdings	152,047	280,644	272,278	−120,231	−44.2	1.7	−3.6

**Fig. 3:**
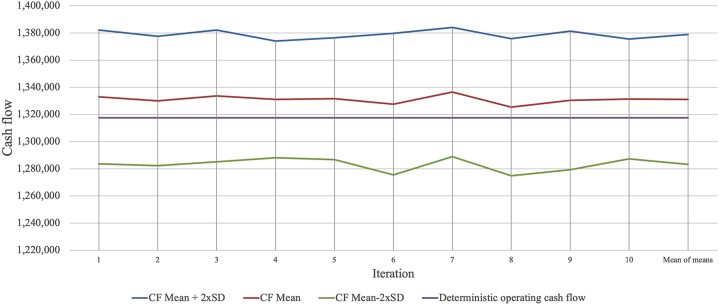
Comparison of the deterministic operating cash flow (1,317,470,10^3^ euros) with the MC cash flow estimates (10^3^ euros) obtained in each iteration and the mean of means. Legend: CF = operating cash flow; SD = standard deviation.

In all iterations of the simulation experiment, the deterministic operating cash flow, as well as the grand mean, is within plus or minus two standard deviations from the mean interval of the operating cash flow distribution (see Fig. 3).

## 5 Discussion and conclusions

According to Peter Drucker, “If you can't measure it, you can't manage it” [63]. Thus, the resilient and sustainable recovery from the Covid-19 pandemic requires the ex-ante substantiated assessment of the extent of the repercussions of the pandemic. The main research purpose of this paper is to contribute to that end. As such, we examine the economic and financial impacts on the performance of the Portuguese mainland hotel industry during the 2020–2021 Covid-19 pandemic period in terms of the industry's aggregate operating revenues, net total assets, net total debt, generated cash flow, and financial slack, using a deterministic approach and stochastic robustness checking. We found that, on average, over the 2020–2021 period, the Covid-19 pandemic inflicted an aggregate impact of: (*i*) a 64.2% reduction in operating revenues (−5317 million euros); (*ii*) a 30.8% decrease in the industry's net total assets (−7625 million euros); (*iii*) a 29.8% increase in the sector's indebtedness (+2557 million euros); (*iv*) a 160.1% decrease in net income (−1457 million euros); (*v*) a 56.0% drop in the operating cash flow (−738 million euros); (*vi*) an 81.1% decline in financial slack (−9443 million euros). Overall, these (deterministic) findings, on the one hand, quantify the extent of the economic problem caused by the pandemic. On the other hand, they provide estimations of the economic thresholds to be overcome and the financial hurdles faced by the future sustainable recovery of the industry.

The robustness check, conducted through the MC simulation with bootstrapping, indicates that the deviations of the deterministic from the stochastic estimates are, at a 95% confidence interval: 1.1% for the operating revenues; 2.1% for the net total assets; 5.5% for the net total debt; 1.9% for the net income; 0.5% for the operating cash flow; and 0.5% for the financial slack.

Overall, not only the stochastic approach delivers comparable outputs for the variables at interest, but unlike the deterministic approach, only the output analysis of the simulation output can be used to understand what happens at the lower tail of the resulting cash flow distribution. In addition, the bootstrap MC approach allowed for an estimate of the uncertainty of the operating cash flow, resulting in an expected downside risk of the Portuguese mainland hotel industry over the 2020–2021 period of 1293 million euros. In other words, such is the maximum shortfall of our industry sample's generated net cash flow during the Covid-19 period. In this framework, the MC model provides more information than the deterministic model and is a valuable tool for assessing the effects of the Covid-19 pandemic on the Portuguese mainland hotel industry.

The 2020 and 2021 impacts on accounting economic aggregates, such as operating revenues, net income, and operating cash flow, were more negative in the first year of the pandemic, as expected. We conjecture that this is due to the initial rounds of government lockdown measures, travel bans, and restrictions. However, the massive vaccination deployment, and the governmental fiscal policy measures to provide emergency countercyclical support to households and firms, may have contributed to the downscaling of the impacts of those effects in 2021 (e.g., Ref. [64]). The financial impacts, measured, for example, by the stocks of net total debt and financial flexibility, reflect, among other factors, the funding needs associated with the negative cash flow generation over the 2020–2021 period, and the effect of the moratoriums on bank credit agreements enacted in March 2020 because of the Covid-19 health emergency.^19^

We can conclude that the extreme uncertainty and volatility associated with events, such as the Covid-19 pandemic, can expose business activity to extremely adverse economic and financial consequences. Our findings are consistent with the claim that was the case in the Portuguese hotel industry.

A limitation of this study is that it focused only on hotels (ORBIS/Sabi databases do not cover very small accommodation units) and treated the sample as a whole without distinguishing hotel size or exploring possible recovery strategies. As such, future research on this topic should develop along two axes. The first is to use a difference-in-differences approach to study whether or not hotels grouped by star classification were impacted differently by the Covid-19 pandemic. The second, building on the conceptual framework that business strategies can be conceptualized as chains of real options, is to develop a randomized valuation framework to appraise the value creation potential of the post-pandemic recovery strategies of the hotel industry.

## Author contribution statement

Mário Coutinho dos Santos, José Magano, Jorge Mota: Conceived and designed the experiments; Performed the experiments; Analyzed and interpreted the data; Contributed reagents, materials, analysis tools or data; Wrote the paper.

## Data availability statement

Data will be made available on request.

## Declaration of competing interest

The authors declare that they have no known competing financial interests or personal relationships that could have appeared to influence the work reported in this paper

## Figures and Tables

**Table:** 

Variable	Specification
*S*	Operating revenue
*C&E*/*S*	Cash & equivalents/Sales
*RCV*/*S*	Receivables/Sales
*INV*/*S*	Inventory/Sales
*OCA*/*S*	Other current assets/Sales
*FAS*/*S*	Fixed assets/Sales
*PAY*/*S*	Payables/Sales
*OCL*/*S*	Other current liabilities/Sales
*NIC*/*S*	Net income/Sales
*r*	Net income - dividends
*D*/*E*	Debt/Equity

**Table:** 

Panel A | Income statement	
Variable	Specification
Operating revenue	Net Sales_*t*_ + Inventory variation_*t*_,
Sales	Net Sales_*t*-1_ x (1 + *g*)
Cost of sales	operating revenues_*t*_ x (cost of sales_2019_/operating revenues_2019_)
Operating costs	Labor Costs_*t*_ + Other Operating Costs_*t*_
Depreciation	Operating revenues_*t*_ x (Depreciation_2019_/Operating revenues_2019_)
EBIT	Operating Revenue - Cost of Sales - Operating Costs - Depreciation
Net interest expense	Interest paid_*t*_ + Incremental Financial Expense_*t*_ – Incremental Financial Revenue_*t*_
P/L before tax	EBIT - Net interest expense
Income taxes	P/L before tax x Income tax rate
Net income	P/L before tax - Income taxes
Paid out dividends	Net income_*t*_ x [Net income_2019_ – (Equity_2019_ - Equity_2018_)]/net income_2019_
Retained earnings	Net income_*t*_ - Paid out dividends_*t*_
Panel B | Balance sheet	
Variable	Specification
Fixed assets	Fixed assets_*t*_/Sales_I_
Current assets	Inventory_*t*_ + Receivables_*t*_ + Other Current Assets_*t*_ + Cash & Equivalents_*t*_
Inventory	Inventory_*t*_/Sales_*t*_
Receivables	Receivables_*t*_/Sales_*t*_
Other current assets	Other Current Assets_*t*_/Sales_*t*_
Cash & equivalents	Operating revenues_*t*_ x Cash & equivalents_2019_/Operating revenues_2019_
TOTAL ASSETS	
Equity	Equity_*t*-1_ + Retained Earnings_*t*_
Liabilities	
Non-current liabilities	
Funding needs	Total Assets_*t*_ – Equity_*t*_ - Non-current Liabilities_*t*_**-** Current Liabilities_*t*_
Current liabilities	Payables_*t*_ + Other Current Liabilities_*t*_
TOTAL EQUITY + LIABILITIES	
Panel C | Cash flow statement	
Variable	Specification
Operating Cash Flow (gross)	Net Interest Expense_*t*_ + Depreciation & Amortization_*t*_
Δ Working capital	Δ Receivables_*t*_ + Δ Inventories_*t*_ - Δ Payables_*t*_
Operating Cash Flow	Operating Cash Flow (gross)_*t*_ - Δ Working capital_*t*_
Panel D | Financial slack	
Variable	Specification
Debt Capacity	(Earnings Before Interest & Taxes_*t*_/Interest Coverage Ratio_*t*_)/(r_*F*_ + Credit Risk Premium
Excess Cash Holdings	Cash & Equivalents_*t*_ – Liquidity Buffer_*t*_
Liquidity Buffer	Defense Interval_*t*_ x Daily Operating Expenses_*t*_
Defense Interval	Current Assets_*t*_/Daily Operating Expenses_*t*_
Financial Slack	Debt Capacity_*t*_ – Non-Current Liabilities_*t*_ + Excess Cash Holdings_*t*_

## Appendix I Sustainable growth model derivation

Following Van Horne (2002), Zantout (1990), and Higgins (1977), we derived a steady-state version of the sustainable growth rate model to estimate the 2020 and 2021 income statements, balance sheets, and cash flow statements for a sample of the Portuguese mainland hotel industry. The model was derived under the assumption that balances of balance sheet accounts are optimized in relation to the current level of sales and that depreciations were not an available source of funds because an application of the same amount in fixed assets is assumed to sustain their operational functionality.

Where C&E denotes cash and equivalents; *S*, operating revenue; *RCV*, receivables; *INV*, inventory; *OCA*, other current assets; *FAS*, net fixed assets; *PAY*, payables; *OCL*, other current liabilities; *NIC*, net income; *D*, Debt; *E*, Equity; *r*, retention rate; *g* denotes sales sustainable growth rate.

$$\begin{array}{l} \frac{C\&E}{S}\Delta S+\frac{RCV}{S}\Delta S+\frac{INV}{S}\Delta S+\frac{OCA}{S}\Delta S+\frac{FAS}{S}\Delta S=r\frac{NIC}{S}\left(S+\Delta S\right)+\left[r\frac{NIC}{S}\left(S+\Delta S\right)\right]\frac{D}{E}+\frac{PAY}{S}\Delta S+\frac{OCL}{S}\Delta S\\ \frac{C\&E}{S}\Delta S+\frac{RCV}{S}\Delta S+\frac{INV}{S}\Delta S+\frac{OCA}{S}\Delta S+\frac{FAS}{S}\Delta S=r\frac{NIC}{S}\left(S+\Delta S\right)\left(1+\frac{D}{E}\right)+\frac{PAY}{S}\Delta S+\frac{OCL}{S}\Delta S\\ \frac{C\&E}{S}\Delta S+\frac{RCV}{S}\Delta S+\frac{INV}{S}\Delta S+\frac{OCA}{S}\Delta S+\frac{FAS}{S}\Delta S=r\frac{NIC}{S}\left(1+\frac{D}{E}\right)S+r\frac{NIC}{S}\left(1+\frac{D}{E}\right)\Delta S+\frac{PAY}{S}\Delta S+\frac{OCL}{S}\Delta S\\ \frac{C\&E}{S}\Delta S+\frac{RCV}{S}\Delta S+\frac{INV}{S}\Delta S+\frac{OCA}{S}\Delta S+\frac{FAS}{S}\Delta S-r\frac{NIC}{S}\left(1+\frac{D}{E}\right)\Delta S-\frac{PAY}{S}\Delta S-\frac{OCL}{S}\Delta S=r\frac{NIC}{S}\left(1+\frac{D}{E}\right)S\\ \Delta S\left[\frac{C\&E}{S}+\frac{RCV}{S}+\frac{INV}{S}+\frac{OCA}{S}+\frac{FAS}{S}-\frac{PAY}{S}-\frac{OCL}{S}-r\frac{NIC}{S}\left(1+\frac{D}{E}\right)\right]=r\frac{NIC}{S}\left(1+\frac{D}{E}\right)S\\ g=\frac{\Delta S}{S}=\frac{r\frac{NIC}{S}\left(1+\frac{D}{E}\right)}{\frac{C\&E}{S}+\frac{RCV}{S}+\frac{INV}{S}+\frac{OCA}{S}+\frac{FAS}{S}-\frac{PAY}{S}-\frac{OCL}{S}-\left[r\frac{NIC}{S}\left(1+\frac{D}{E}\right)\right]} \end{array}$$

## Footnotes

1 According to Instituto Nacional de Estatística [1], the direct and indirect contribution of the tourism industry to GDP dropped from 11.8% in 2019 to 6.6% in 2020 and rose to 8.0% in 2021 (see also [[2], [3], [4]]).

2 For further details on the pandemic's impacts on the tourism industry, see, e.g., Refs. [[15], [16], [17], [18], [19], [20], [21], [22], [23], [24]].

3 Our deterministic estimations incorporate the Covid-19 mitigating measures decided at the domestic government level, within the scope of the policies implemented at the European Union level, to support the tourism sector during the pandemic period. Measures include, but are not limited to, credit lines, flexibility in tax payment, and the deferral of the payment of social benefits (Portuguese versions are available at: https://www.bportugal.pt/sites/default/files/anexos/nota_sobre_as_principais_medidas_covid19.pdf; https://www.portugal.gov.pt/pt/gc22/comunicacao/comunicado?i=governo-adota-medidas-para-apoiar-empresas-e-trabalhadores; https://www.portugal.gov.pt/pt/gc22/comunicacao/noticia?i=governo-toma-medidas-extraordinarias-para-responder-a-epidemia-de-covid-19; http://business.turismodeportugal.pt/pt/Gerir/covid-19/Paginas/medidas-de-apoio-economia.aspx; all accessed on October 5, 2022).

4 'Risk' and 'uncertainty' are often used interchangeably. However, as insightfully articulated by Frank H. Knight in 1921 [30], the two concepts have distinct meanings: risk relates to objective probabilities, whereas uncertainty relates to subjective probabilities (see also [31,32]).

5 See Appendix I for the derivation of a steady state pre-pandemic version of the sustainable growth rate model, derived to estimate the financial income statements for 2020 and 2021.

6 Henceforth, we use ‘financial flexibility’ and ‘financial slack’ interchangeably.

7 See Panel D of Appendix II. It should be noted that cash holding balances are firm-specific and determined by firm characteristics, such as size, cash flow generation profile, growth opportunities set, and firm positioning in its life cycle.

8 Conceptually, *CFaR* is a metric similar to value-at-risk (*VaR*), which “summarizes the worst loss over a target horizon that will not be exceeded with a given level of confidence” [52]. Specifically, *VaR* describes the quantile of the projected distribution of gains and losses over a target horizon. Specifying *c* as the selected confidence level, *VaR* corresponds to the *1-c* lower tail level. Unlike *CFaR*, *VaR* does not include the time value of money because the short time horizon does not require periodic cashflows to be discounted [26].

9 See Ref. [59] for a comprehensive description of the use of the MC approach in risk analysis.

10 See, Appendix I for the derivation of the sustainable growth rate model.

11 Test results are available from the corresponding author upon request.

12 The National Register of Tourism Enterprises (RNET) provides a database on tourism firms with a valid license, including data on, e.g., firm denomination, type of tourism unit, capacity, location, and age: http://business.turismodeportugal.pt/pt/Planear_Iniciar/Licenciamento_Registo_da_Atividade/Empreendimentos_Turisticos/Paginas/rnet.aspx.

13 Data on the sampled firms for the year 2021 were drawn from the Orbis/Sabi databases on September 30, 2022, when only 915 of the 972 sample firms had disclosed their data to the database. Therefore, we estimate 2021 financial statements as a direct proportion, using 915/972 as a constant of proportionality.

14 Data available at: http://pages.stern.nyu.edu/∼adamodar/New_Home_Page/dataarchived.html.

15 The “Liquidity Buffer” (the minimum cash balance requirement) is specified as the product of the 2019 defensive interval ratio by the daily operational expenses.

16 The Monte Carlo simulation was carried out using the “Oracle Crystal Ball” add-in to MS-Excel, release 11.1.2.4.900 (64-bits).

17 The grand mean of the ten independent and identically distributed simulations is similar, under the linearity assumption, to generating ten samples from one simulation only. Nonetheless, the greater the number of simulation trials we run, the more the resulting means distribution will tend towards the true output variable distribution.

18 Detailed results are available from the authors upon reasonable request from registered Oracle Crystal Ball (64-bits) licensees.

19 See Decree of Law No. 10 J/2020 of 26 March 2020; https://www.bportugal.pt/en/comunicado/covid-19-moratorium-credit-agreements-bank-customers-entered-force, accessed on October 24, 2022.
