# Supplementary material for: The impact of the Covid-19 pandemic on the hotel Industry’s economic performance: Evidence from Portugal
Source: Heliyon. 2023 May 3;9(5):e15850. doi: 10.1016/j.heliyon.2023.e15850 (PMC10154056; doi:10.1016/j.heliyon.2023.e15850)
Supplement: Multimedia component 1 [file mmc1.pdf]

Ouriginal
by Turnitin

Document Information

**Analyzed document**	Portuguese-Hotel-Industry_11__.pdf (D164631701)
**Submitted**	4/21/2023 10:12:00 AM
**Submitted by**	José Magano
**Submitter email**	jmagano@ua.pt
**Similarity**	0%
**Analysis address**	jmagano.ua@analysis.urkund.com

Sources included in the report

W	URL: https://blogs.worldbank.org/education/you-can-t-manage-what-you-don-t-measure<br>Fetched: 4/21/2023 10:12:00 AM	1
W	URL: https://wttc.org/news-article/portugals-travel-and-tourism-could-surpass-pre-pandemic-levels-in-2023<br>Fetched: 4/21/2023 10:12:00 AM	1
SA	**FELLYA (34035707) BUS308 TSA 2021 Final Exam Nov.pdf**<br>Document FELLYA (34035707) BUS308 TSA 2021 Final Exam Nov.pdf (D121307140)	3

Entire Document

Ouriginal
by Turnitin

1 The Impact of the Covid-19 Pandemic on the Hotel Industry's Economic Performance: Evidence from Portugal* Mário Coutinho dos Santos, a José Magano b and Jorge Mota c a CICEE, Research Center in Economics & Business Sciences, Portugal, and Catholic University of Portugal. b CICEE, and Higher Institute of Business Sciences and Tourism (ISCET), Portugal. c CICEE, DEGEIT, University of Aveiro, and GOVCOPP, Portugal. Abstract This paper estimates the impact of the Covid-19 pandemic on the economic and financial performance of the Portuguese mainland hotel industry. For that purpose, we implement a novel empirical approach to gauge the impact of the pandemic during the 2020-2021 period in terms of the industry's aggregated operating revenues, net total assets, net total debt, generated cash flow, and financial slack. To that end, we derive and estimate a sustainable growth model to project the 2020 and 2021 'Covid-free' aggregated financial statements of a representative Portuguese mainland hotel industry sample. The impact of the Covid pandemic is measured by the difference between the 'Covid-free' financial statements and the historical data drawn from the Orbis and Sabi databases. An MC simulation with bootstrapping indicates that the deviations of the deterministic from the stochastic estimates for major indicators vary between 0.5 and 5.5 percent. The deterministic operating cash flow estimate lies within plus or minus two standard deviations from the mean interval of the operating cash flow distribution. Based on this distribution, we estimate the downside risk, measured by cash flow at risk, at 1,294 million euros. Overall findings shed some light on the economic and financial repercussions of extreme events such as the Covid-19 pandemic, providing us with a better understanding of how to design public policies and business strategies to recover from such an impact. Key words: cash-flow at risk; hotel industry; Covid-19; Monte Carlo simulation JEL codes: L83, Z32, L25, C63 * Authors gratefully acknowledge the useful and valuable comments and suggestions from Celeste Eusébio, Fernando Tavares, Paulo Alves, and Rui Neves on an earlier draft, and research seminar participants at CICEE – Research Center in Economics & Business Sciences, Portugal (October 2022). We are thankful for helpful comments from an anonymous editor and two anonymous reviewers. We also thank GOVCOPP at the University of Aveiro for providing access to AMADEUS/ORBIS and SABI databases, and INE (Statistics Portugal) for providing access to the "Sistema de Contas Integradas das Empresas" (SCIE) database. The authors are solely responsible for any inexactitudes, omissions, and errors. This paper is part of the research program "PTDC/EGE- ECO/4993/2021 - THE PORTUGUESE HOTEL INDUSTRY: PREPARING FUTURE AHEAD OF COVID-19 PANDEMIC" submitted to Fundação para a Ciência and Tecnologia (FCT Portugal), which did not receive any specific grant from funding agencies in the public, commercial, or not-for-profit sectors.

2 1. Introduction The outbreak of the Covid-19 pandemic (hereafter, referred to as the 'pandemic') triggered unprecedented global disruptions in tourism and hospitality ecosystems and these sectors plunged into a severe economic and financial crisis. Unsurprisingly, such economic, financial and social impacts have a greater negative impact on economies like that of Portugal, which are much more dependent on the performance of the tourism sector than other countries. 1 At the global hotel industry level, most have been forced to downsize their operations, resulting in significant economic imbalances. Ultimately, some of them may have been driven into financial distress or even insolvency. For example, the 11.1 percent operating revenue average annual growth rate of the Portuguese mainland hotel industry during the 2014-2019 period was abruptly interrupted by pandemic lockdown measures, travel bans, and other restrictions, triggering unprecedented disruptions and plunging the sector into economic turmoil, with grievous social-economic externalities [1]. Notably, it led to a generalized scale- down of hotel operations, plummeted revenues, fueled workforce layoff, and exposed the ecosystem to economic and financial disarray [5-7]. Although prospects for the recovery of the tourism sector remained relatively unsteady, governmental policymakers and hotel ecosystem participants alike strove to map out alternative feasible recovery paths (and pace) for designing post-pandemic resilient and sustainable recovery strategies for the industry (e.g., [8]). The most recent research on the topic examines either public policy aimed at mitigating the impact of COVID-19 on tourism (e.g., [9-11]), or explores particular performance dimensions of specific segments of the hotel industry, such as listed hotels (e.g., [12-15]). 2 Yet, the development of blueprints to support the design of prospective strategic scenarios requires the availability of a comprehensive and quantitative assessment of the magnitude of the economic, financial, and social impacts of the Covid-19 shock. To our knowledge, no such assessment has been carried out, motivating us to perform this study. The main research objective of this paper is to quantitatively assess the potential economic and financial shocks on the performance of the Portuguese hotel industry. Data are based on approximately 1,000 hotels in mainland Portugal in the period 2020-2021. Besides providing evidence of the aggregated pandemic's impact on the economic and financial condition of the industry, this work also contributes to the literature by applying a novel methodological approach. First, it derivates and estimates a deterministic business model 1 According to Instituto Nacional de Estatística [1], the direct and indirect contribution of the tourism industry to GDP dropped from 11.8 percent in 2019 to 6.6 percent in 2020 and rose to 8.0 percent in 2021 (see also, [2-4]). 2 For further details on the pandemic's impacts on the tourism industry, see, e.g., [16-25].

Ouriginal by Turnitin

3 for the sector, anchored on the maximum sustainable growth rate (SGM) conceptual framework to gauge the impact of the pandemic during the 2020-2021 period in terms of operating revenues, net total assets, net total debt, operating cash flow, and financial slack. 3 Next, this approach combines with a robustness check that incorporates stochastic variability into the deterministic base case by applying Monte Carlo (MC) methods with bootstrapping to measure the downside risk of our hotel industry sample using the cash-flow-at-risk (CFaR) conceptual framework. 2. Conceptual background 2.1 Maximum sustainable growth framework Tourism and hospitality industries are exposed to a wide spectrum of risks. Therefore, enterprise risk management (ERM) — risk identification, measurement, and management — is instrumental in helping firms to manage their value creation objectives, particularly in terms of mitigating financial distress and optimizing risk portfolio (e.g., [26-30]). 4 At the hotel firm level, the measurement of the expected impact of downside risk factors on value creation should be a primary managerial concern (e.g., [28, 34-36]. Further, there is abundant and compelling evidence that the volatility in corporate accounting aggregates, such as net income and operating cash flows, is related to value creation (e.g., Smithson & Simkins, 2005 [34]). Under well-diversified firm ownership, risk management can be expected to be positively related to a firm's value, which could limit the expected costs of financial distress, manage financial slack, reduce tax liability, and mitigate suboptimal resource allocation (e.g., [35]). To estimate the economic and financial impact of the Covid-19 pandemic on the Portuguese mainland hotel industry in 2020 and 2021, we develop a novel empirical, methodological approach based on the SGM framework. SGM builds on the percentage-of- sales method's standard assumptions that the stocks of the balance sheet accounts are optimized 3 Our deterministic estimations incorporate the Covid-19 mitigating measures decided at the domestic government level, within the scope of the policies implemented at the European Union level, to support the tourism sector during the pandemic period. Measures include, but are not limited to, credit lines, flexibility in tax payment, and the deferral of the payment of social benefits (Portuguese versions are available at: https://www.bportugal.pt/sites/default/files/anexos/nota_sobre_as_principais_medidas_covid19.pdf; https://www.portugal.gov.pt/pt/gc22/comunicacao/comunicado?i=governo-adota-medidas-para-apoiar- empresas-e-trabalhadores; https://www.portugal.gov.pt/pt/gc22/comunicacao/noticia?i=governo-toma-medidas- extraordinarias-para-responder-a-epidemia-de-covid-19; http://business.turismodeportugal.pt/pt/Gerir/covid- 19/Paginas/medidas-de-apoio-economia.aspx; all accessed on October 5, 2022). 4 'Risk' and 'uncertainty' are often used interchangeably. However, as insightfully articulated by Frank H. Knight in 1921 [31], the two concepts have distinct meanings: risk relates to objective probabilities, whereas uncertainty relates to subjective probabilities (see also [32,33]).

4 in relation to the current level of sales and vary in proportion to sales; and that depreciation and amortization are not an available source of funds because it is assumed that the same amount is applied in restoring fixed assets operational functionality (e.g., [37-39]). 5 Under the maximum annual percentage increase in operating revenue — g — a firm can sustain, keeping constant at the pre-pandemic levels (2019): (i) fixed assets utilization, proxied by the net fixed assets-to-operating revenue ratio; (ii) after-tax operating revenue profitability, measured by the net income-to-operating revenue ratio; (iii) capital structure gauged by the debt-to-equity ratio; and (iv) the retention rate of earnings, measured by the complement of the dividend payout ratio, and without resorting to incremental external funding. Financial slack (FS) is a readily available liquidity cushion in the form of excess cash holdings and debt capacity, which provides financing flexibility by mitigating the impact of adverse liquidity shocks, and financial distress and by moderating suboptimal allocative behavior, namely in the form of underinvestment (e.g., [13]). 6 Therefore, for precautionary reasons, firms tend to accumulate liquid assets, such as cash and equivalents, as an 'insurance' against liquidity shortfalls arising in adverse states of cash flow generation and to avoid asset fire sales, raising externally costly unanticipated funding, or incurring inefficient underinvestment (e.g., [40-43]). Under this framework, firms with higher asset systematic riskiness and costlier access to external capital markets tend to carry larger cash holdings on their balance sheets. We measure excess cash holdings as the difference between "Cash & Equivalents" and the "Liquidity Buffer" balances (e.g., [44-47]). 7 Conceptually, debt capacity is the incremental borrowing required to sustain the capital market's perception of a firm's current aggregate asset systematic riskiness. Or restated, the maximum amount that could optimally be borrowed at the current risk-adjusted marginal cost of debt (e.g., [40, 48, 49]). 2.2 Cash-flow-at-risk Another valuable tool to assess non-financial firms' downside risk is cash flow at risk (CFaR). CFaR is a composite measure of the maximum decrease in expected cash flows associated with the uncertainty of risk factors, given a pre-defined confidence level, for a given 5 See Appendix I for the derivation of a steady state pre-pandemic version of the sustainable growth rate model, derived to estimate the financial income statements for 2020 and 2021. 6 Henceforth, we use 'financial flexibility' and 'financial slack' interchangeably. 7 See Panel D of Appendix II. It should be noted that cash holding balances are firm-specific and determined by firm characteristics, such as size, cash flow generation profile, growth opportunities set, and firm positioning in its life cycle.

Ouriginal by Turnitin

5 period, which Stein et al. [50] define "as the probability distribution of a company's operating cashflows over some horizon in the future, based on information available today". Taking a prespecified timeframe and statistical confidence level, the CFaR approach to downside risk measurement estimates the maximum shortfall of cash a firm is willing to accept and, therefore, its overall liquidity risk over a given period (e.g., [28, 50-52]. 8 Moreover, since all risk exposures can be aggregated into a single metric, CFaR provides quantitative information, at least accurate on average, helping to guide managerial decision-making (e.g., [35, 54, 55]). Indeed, 'it is the "lower tail" of the cash flow distribution that can have costly consequences, such as insufficient funds to carry out the company's investment program or even bankruptcy' [55]; CFaR provides a measure of such lower tail effects, which we estimate by bootstrapping the Portuguese mainland hotel industry model for robustness-checking purposes, following Alexander [56]. It should be emphasized that the data panel used in the deterministic methodological approach features an inherent statistical significance limitation, which inhibits inferential testing. The estimation of CFaR with Monte Carlo (MC) simulation has been addressed in the literature, namely, through examples that suggest that this numerical tool is effective for solving problems in finance that involve closed-form analytical solutions that are too complex or impossible to determine (e.g., [57-58]). Besides the benefit of efficiently dealing with complexity, another advantage of MC is its inherent randomness, which is essential for simulating real-life random systems [59]. This method is, therefore, an obvious choice for tackling the CFar estimation as a robust check in our study's approach. Hence, we use MC bootstrapping computational methods to perform the number of trials necessary in each simulation experiment to generate a numerical approximation to the true distribution of the output variable at the standard 95 percent confidence level. 9 3. Methods 3.1 Research design This paper examines the impact of the Covid-19 pandemic period during the 2020 and 2021 sampling periods on the Portuguese mainland hotel industry. Specifically, we estimate 8 Conceptually, CFaR is a metric similar to value-at-risk (VaR), which "summarizes the worst loss over a target horizon that will not be exceeded with a given level of confidence" [53] . Specifically, VaR describes the quantile of the projected distribution of gains and losses over a target horizon. Specifying c as the selected confidence level, VaR corresponds to the 1-c lower tail level. Unlike CFaR, VaR does not include the time value of money because the short time horizon does not require periodic cashflows to be discounted [27] . 9 See [60] for a comprehensive description of the use of the MC approach in risk analysis.
6 the pandemic effect in terms of total net assets, total net debt, operating cash flow, and financial flexibility, for a representative sample of Portugal's mainland hotels (hereafter, referred to as the 'sample'). The empirical implementation strategy is designed in five steps. First, we estimate aggregate balance sheets, income, and operating cash flow statements for the 2014-2021 sampling period, drawing data from the Orbis/Sabi databases. Second, we derive a steady-state version of SGM and estimate the operating revenue sustainable growth rate to forecast the sample's financial statements for 2020 and 2021. 10 Regarding the third step, we project 2020 and 2021 aggregate balance sheets, income statements and cash flow statements, which are unconditioned by the occurrence of the Covid-19 pandemic. Fourth, we measure the (deterministic) impact of the Covid-19 pandemic as the difference between the projected and observed 2020's and 2021's aggregated operating revenues, net total assets, total net debt, operating cash flow, and financial slack. Fifth, we run a Monte Carlo simulation experiment to check for the robustness of the deterministic 2019 base case in terms of the output variables required to compute the CFaR. 3.2 Data Economic, financial, and operating data for this research were drawn from INE (Statistics Portugal), Sabi, and Orbis, covering the 2010-2021 sampling period. However, to minimize the spillovers of the financial assistance program signed between Portugal and the International Monetary Fund, the European Union, and the European Central Bank, encompassing the application of a three-year economic adjustment program (2011-mid-2014), we restricted the sampling period to 2014-2019. Results of summary statistics and parametric tests for equality of means document that the variables used in the deterministic model do not exhibit, at the standard confidence levels, statistically significant differences in means between the sampling subperiods of 2010-2019 and 2014-2019. These results support the consistency of using the 2014-2019 subsampling period for this empirical analysis and segmenting hotels by star category. 11 To be included in the sample, a hotel must comply with the following criteria: (i) be included in the National Register of Tourism Enterprises (RNET) database, with an assigned fiscal number; (ii) be established and operating on Portugal's mainland (iii) be a star classified 10 See, Appendix I for the derivation of the sustainable growth rate model. 11 Test results are available from the corresponding author upon request.

Ouriginal
by Turnitin

7 hotel or hotel-apartment; (iv) be active for the entire sampling period. 12 The population of hotels and hotel-apartments was drawn from the INE database for the 2019-2021 period (Table 1). >INSERT TABLE 1 HERE< A search in the RNET database yielded a sample of 1,282 hotel units with assigned fiscal numbers and star classifications that met the above-mentioned criteria. Finally, we identified 972 entities in Orbis/Sabi databases that matched that set and reported complete data for the 2019-2021 period, which corresponded to 1,057 hotel and hotel-apartment units. For this sample, we extracted economic, financial, and operating data at the hotel business firm level (Table 1). 13 Data on income statement items, such as labor costs, other operating costs, financial revenues, and financial expenses, were drawn from the Sabi database. In addition, data on the interest coverage ratios and the debt spreads associated with the synthetic credit ratings were collected from Prof. Aswath Damodaran's website; 14 10-year government bond yields for Portugal and triple-A rating countries were collected from the European Central Bank's - Statistical Data Warehouse. 3.3 SGM modeling and estimation Aggregate balance sheets, income, and cash flow statements were estimated using the variable specified in Appendix II. Financial slack is modeled as the sum of excess cash holdings (ECH) and debt capacity (DC). ECH is measured as the difference between the book value of cash and equivalents, and the minimum cash balance requirements (e.g., [45]). 15 We use the defensive interval ratio (DIR) to estimate the short-term liquidity needs in terms of the number of days a hotel could operate resorting only to its current assets (e.g., [61]): 12 The National Register of Tourism Enterprises (RNET) provides a database on tourism firms with a valid license, including data on, e.g., firm denomination, type of tourism unit, capacity, location, and age:
http://business.turismodeportugal.pt/pt/Planear_Iniciar/Licenciamento_Registo_da_Atividade/Empreendimentos_Turisticos/Paginas/rnet.aspx. 13 Data on the sampled firms for the year 2021 were drawn from the Orbis/Sabi databases on September 30, 2022, when only 915 of the 972 sample firms had disclosed their data to the database. Therefore, we estimate 2021 financial statements as a direct proportion, using 915/972 as a constant of proportionality. 14 Data available at: http://pages.stern.nyu.edu/~adamodar/New_Home_Page/dataarchived.html. 15 The "Liquidity Buffer" (the minimum cash balance requirement) is specified as the product of the 2019 defensive interval ratio by the daily operational expenses.

8 (1) where current assets include cash, marketable securities, and net receivables; and daily operational expenses are measured by the sum of the cost of sales, operating costs, and net interest rate divided by 360. We define DC as the maximum amount of borrowing lenders are willing to extend to an investment-grade rated firm based on its interest coverage ratio and the debt spread associated with its rating notation (see, e.g., [62]). Hotel's DC is specified as: (2) where r F denotes the rate of return on a riskless asset; and spread, the debt's default risk premium. We estimate hotel synthetic rating notations, interest coverage ratios, and the debt default spreads associated with them using Aswath Damodaran's website [28] (Table 2). The model uses the operating income (EBIT) and the net interest expense as inputs to estimate the interest coverage ratio, which is extensively used by Standard and Poor's and Moody's, two leading international rating agencies. >INSERT TABLE 2 HERE< 3.4 MC approach We use MC computational numerical methods with bootstrapping to produce forecasts of the 2020 and 2021 aggregated operating revenues, net total assets, net total debt, operating cash flow, financial slack, debt capacity, and excess cash holdings to check for the robustness of the deterministic model. A single independent variable — operating revenues sustainable growth rate — is randomized, specified as a triangular distribution, and heuristically calibrated as follows: minimum = 0 percent; likeliest value = 12 percent; and maximum = 17 percent. 16 The MC simulation follows a bootstrap multiple-simulation approach, repeatedly simulating the model and then creating a distribution of the statistics from each simulation, as depicted in Figure 1. 16 The Monte Carlo simulation was carried out using the "Oracle Crystal Ball" add-in to MS-Excel, release 11.1.2.4.900 (64-bits). t t Current Assets DIR Daily Operating Expenses = & t t t F Earnings Before Interests Taxes Interest Coverage Ratio Debt Capacity r spread = +

9 Figure 1 – Bootstrap multiple-simulation method Source: Crystal Ball User Guide. 4. Results 4.1 SGM estimation Data in Table 1 document that during the Covid-19 shock, the hotel population diminished relatively to 2019. However, it only partially recovered in 2021 without achieving pre-pandemic levels. We estimate the 2019 'Covid-19-free' operating revenue sustainable growth rate, using the steady-state SGM model derived in Appendix I, at 12.0 percent (see Table 3). We used this growth rate to project pro forma aggregate balance sheets, income, and cash flow statements for the 2020 and 2021 periods (see Appendix II for the specification of the variables). >INSERT TABLE 3 HERE< Panels A, B, and C of Table 4 present the estimation of the sample's aggregate financial statements for the 2020 and 2021 periods, based on the 2019 period's SGR estimate unconditioned by the Covid-19 outbreak. The table presents the 2019 base case (column [1]); the deterministic estimations for 2020 and 2021 aggregate pro forma balance sheet, income, and cash flow statements (columns [2] and [3]); the 2020 and 2021 real aggregate balance sheet, income, and cash flow statements (columns [4] and [5]); and the estimates of the impact of the Covid-19 pandemic on the output variables measured by the differences between the 2020-2020 and 2021-2021 real aggregate values and the deterministic estimates (columns [6] and [7]). >INSERT TABLE 4 HERE<

Ouriginal
by Turnitin

10 The deterministically estimated aggregate impacts inflicted on our Portuguese mainland hotel industry sample over the 2020-2021 pandemic period are presented in Table 5: >INSERT TABLE 5 HERE< The estimated economic impacts measured by the aggregated operating revenues, net income, and operating cash flow are: a 64.2 percent reduction (-5,317 million euros); a 160.1 percent decrease (-1,457 million euros); and a 56.0 percent drop (-738 million euros), respectively. The financial repercussions, gauged by the variation in the non-current liabilities and the financial slack, are: a 29.9 percent increase (+2,557 million euros); and an 87.1 percent decline (-9,316 million euros), respectively. The latter impact is the compound effect of the 53.1 percent fall in debt capacity (-9,444 million euros) and the 45.8 percent reduction in the excess cash holdings (-129 million euros). Despite the reported aggregate impacts of 2020 and 2021, we must recognize that, after a severe decrease in hotel demand in 2020, some recovery was seen in 2021. Accordingly, our model presents different impact variations between the estimated and real outcomes from 2020 to 2021, of which it is worth mentioning the following (i) a 50 million euro increase in operating revenues (1.9 percent); (ii) a 1,238 million euro drop in the industry's net total assets (69.4 percent); (iii) an 894 million euro increase in the sector's non-current liabilities balance (107.4 percent); (iv) a 440 million euro increase in net income (46.4 percent); (v) a 369 million euro increase in the operating cash flow (44.4 percent); (vi) a 4,144 million euro drop in financial slack (344.5 percent); (vii) a 4,998 million euro drop in financial slack (224.9 percent); and (viii) a 4 million euro drop in financial slack (6.6 percent). To summarize, in 2021, net income and operating cash flow improved due to growing demand, whereas net total assets, net total debt, and financial slack worsened (Figure 2). Figure 2 – The impact of the Covid-19 pandemic: 2020, 2021, and 2020-21 (unit: 10 3 euros).

11 The launch of the vaccination campaign by the end of 2020 may have, to a certain extent, lessened tourist travel restrictions, creating the conditions necessary for some recovery in hotel occupancy rates in 2021 and, consequently, in operating revenues. However, as shown in Figure 2, the difference between 2020 and 2021 operating revenues is relatively marginal. We conjecture that this might have been the effect of the phasing out in 2021 of the governmental Covid-19 mitigating measures in place. Debt capacity and excess cash holdings are the two sources of financial flexibility required to mitigate potential liquidity shortfalls and suboptimal allocative behavior in adverse states of the world, such as during the Covid-19 outbreak (See Panel D in Appendix II for the specification of the variables). Results document that over the 2020-2021 period, the hotel industry suffered a major fall-off in financial flexibility due, first and foremost, to debt capacity contraction, which seriously curtails potential recovery funding needs, notably in the new context of central banking's monetary tightening policy. The breakdown of financial slack indicates that borrowing capacity is its main determinant (see Table 5 and Figure 2). To sharpen the analysis of the results, we estimate the 2020 and 2021 impacts of the output variables at the hotel unit level (see Table 6). As expected, the impacts on accounting economic aggregates, such as operating revenues, net income, and operating cash flow, were more negative in the first year of the pandemic. >INSERT TABLE 6 HERE< 4.2 Robustness check: Monte Carlo simulation As previously described, we followed a bootstrap MC multiple-simulation approach, repeatedly running the model. As such, ten simulation experiments were performed, each one with the number of trials required to generate a numerical approximation to the distribution of the output variables. 17 Results are summarized in Table 7. 18 >INSERT TABLE 7 HERE< In each experiment, cash flow at risk was estimated as the operating cash flow for which the accumulated probability of occurrence is 5 percent, or, in other words, the likelihood of 17 The grand mean of the ten independent and identically distributed simulations is similar, under the linearity assumption, to generating ten samples from one simulation only. Nonetheless, the greater the number of simulation trials we run, the more the resulting means distribution will tend towards the true output variable distribution. 18 Detailed results are available from the authors upon reasonable request from registered Oracle Crystal Ball (64- bits) licensees.

12 exceeding CFaR is 95 percent. The Monte Carlo simulation results are very close to those obtained with the deterministic approach described in section 4. Table 8 exhibits the robustness check estimates performed using Monte Carlo simulation methods with bootstrapping for the net total assets, net total debt, operating cash flow, and financial slack at risk, as well as the aggregate impact of the Covid-19 pandemic for the 2020-21 period. These suggest that, on average, the absolute deviations between deterministic and stochastic estimates at 95 percent confidence level are relatively minor, oscillating between 0.5 and 5.5 percent. >INSERT TABLE 8 HERE< In all iterations of the simulation experiment, the deterministic operating cash flow, as well as the grand mean, is within plus or minus two standard deviations from the mean interval of the operating cash flow distribution (see Figure 3). Figure 3 – Comparison of the deterministic operating cash flow (1,317,470 10 3 euros) with the MC cash flow estimates (10 3 euros) obtained in each iteration and the mean of means. Legend: CF = operating cash flow; SD = standard deviation. 5. Discussion and Conclusions According to

92% MATCHING BLOCK 1/5 W

Peter Drucker, “If you can't measure it, you can't manage it” [63].

Ouriginal
by Turnitin

Thus, the resilient and sustainable recovery from the Covid-19 pandemic requires the ex-ante substantiated assessment of the extent of the repercussions of the pandemic. The main research purpose of this paper is to contribute to that end. As such, we examine the economic and financial impacts on the performance of the Portuguese mainland hotel industry during the 2020-2021 Covid-19 pandemic period in terms of the industry's aggregate operating revenues, net total assets, net total debt, generated cash flow, and financial slack, using a deterministic approach and stochastic robustness checking. We found that, on average, over the 2020-2021 period, the Covid-19 pandemic inflicted an aggregate impact of: (i) a 64.2 percent reduction
13 in operating revenues (-5,317 million euros); (ii) a 30.8 percent decrease in the industry's net total assets (-7,625 million euros); (iii) a 29.8 percent increase in the sector's indebtedness (+2,557 million euros); (iv) a 160.1 percent decrease in net income (-1,457 million euros); (v) a 56.0 percent drop in the operating cash flow (-738 million euros); (vi) an 81.1 percent decline in financial slack (-9,443 million euros). Overall, these (deterministic) findings, on the one hand, quantify the extent of the economic problem caused by the pandemic. On the other hand, they provide estimations of the economic thresholds to be overcome and the financial hurdles faced by the future sustainable recovery of the industry. The robustness check, conducted through the MC simulation with bootstrapping, indicates that the deviations of the deterministic from the stochastic estimates are, at a 95 percent confidence interval: 1.1 percent for the operating revenues; 2.1 percent for the net total assets; 5.5 percent for the net total debt; 1.9 percent for the net income; 0.5 percent for the operating cash flow; and 0.5 percent for the financial slack. Overall, not only the stochastic approach delivers comparable outputs for the variables at interest, but unlike the deterministic approach, only the output analysis of the simulation output can be used to understand what happens at the lower tail of the resulting cash flow distribution. In addition, the bootstrap MC approach allowed for an estimate of the uncertainty of the operating cash flow, resulting in an expected downside risk of the Portuguese mainland hotel industry over the 2020-2021 period of 1,293 million euros. In other words, such is the maximum shortfall of our industry sample's generated net cash flow during the Covid-19 period. In this framework, the MC model provides more information than the deterministic model and is a valuable tool for assessing the effects of the Covid-19 pandemic on the Portuguese mainland hotel industry. The 2020 and 2021 impacts on accounting economic aggregates, such as operating revenues, net income, and operating cash flow, were more negative in the first year of the pandemic, as expected. We conjecture that this is due to the initial rounds of government lockdown measures, travel bans, and restrictions. However, the massive vaccination deployment, and the governmental fiscal policy measures to provide emergency countercyclical support to households and firms, may have contributed to the downscaling of the impacts of those effects in 2021 (e.g., [64]). The financial impacts, measured, for example, by the stocks of net total debt and financial flexibility, reflect, among other factors, the funding needs associated with the negative cash flow generation over the 2020-2021 period, and the effect of
14 the moratoriums on bank credit agreements enacted in March 2020 because of the Covid-19 health emergency. 19 We can conclude that the extreme uncertainty and volatility associated with events, such as the Covid-19 pandemic, can expose business activity to extremely adverse economic and financial consequences. Our findings are consistent with the claim that that was the case in the Portuguese hotel industry. A limitation of this study is that it focused only on hotels (ORBIS/Sabi databases do not cover very small accommodation units) and treated the sample as a whole without distinguishing hotel size or exploring possible recovery strategies. As such, future research on this topic should develop along two axes. The first is to use a difference-in-differences approach to study whether or not hotels grouped by star classification were impacted differently by the Covid-19 pandemic. The second, building on the conceptual framework that business strategies can be conceptualized as chains of real options, is to develop a randomized valuation framework to appraise the value creation potential of the post-pandemic recovery strategies of the hotel industry. 19 See Decree of Law No. 10 J/2020 of 26 March 2020; https://www.bportugal.pt/en/comunicado/covid-19- moratorium-credit-agreements-bank-customers-entered-force, accessed on October 24, 2022.
15 References [1] INE Statistics Portugal, Conta Satélite do Turismo para Portugal, (2022). https://www.ine.pt/xportal/xmain?xpid=INE&xpgid=ine_destaques&DESTAQUESdest_boui=54 0831279&DESTAQUESmodo=2 (accessed January 17, 2023). [2] World Travel & Tourism Council, Portugal's

65%	MATCHING BLOCK 2/5	W

Travel & Tourism Could Surpass Pre-Pandemic Levels in 2023, (2022). https://wttc.org/news-article/portugals-travel-and-tourism-could-surpass- pre-pandemic-levels-in-2023 (

accessed December 17, 2022). [3] C. Costa, The impact of the COVID-19 outbreak on the tourism and travel sectors in Portugal: Recommendations for maximising the contribution of the European Regional Development Fund (ERDF) and the Cohesion Fund (CF) to the recovery, Report Produced to the Directorate-General Regional and Urban Policy (DG REGIO), European Commission. (2021). [4] F. Almeida, O. Silva, The impact of COVID-19 on tourism sustainability: Evidence from Portugal, Advances in Hospitality and Tourism Research (AHTR). 8 (2020) 440–446. [5] INE Statistics Portugal, Estatísticas do Turismo 2019, Lisbon, Portugal, 2020. [6] World Travel & Tourism Council, Portugal 2020 Annual Research: Key Highlights, (2021). https://wttc.org/research/economic-impact (accessed January 17, 2023). [7] INE Statistics Portugal, Estatísticas do Turismo 2020, Lisbon, Portugal, 2021. [8] A. Fotiadis, S. Polyzos, T.-C.T. Huan, The good, the bad and the ugly on COVID-19 tourism recovery, Annals of Tourism Research. 87 (2021) 103117. doi: 10.1016/j.annals.2020.103117. [9] D. Le, G. Phi, Strategic responses of the hotel sector to COVID-19: Toward a refined pandemic crisis management framework, International Journal of Hospitality Management. 94 (2021) 102808. doi: 10.1016/j.ijhm.2020.102808. [10] T. Tressel, D. Xiaodan, Global Corporate Stress Tests-Impact of the COVID-19 Pandemic and Policy Responses, SSRN. (2021). [11] Organisation for Economic Co-operation Development, Mitigating the impact of COVID-19 on tourism and supporting recovery, OECD Tourism Papers. (2020). [12] L. Silva, The impact of the COVID-19 pandemic on rural tourism: a case study from Portugal, Anatolia. 33 (2022) 157–159. doi: 10.1080/13032917.2021.1875015. [13] X. Zhang, B.-G. Chang, K.-S. Wu, COVID-19 Shock, Financial Flexibility, and Hotels' Performance Nexus, Frontiers in Public Health. 10 (2022) 792946. doi: 10.3389/fpubh.2022.792946. [14] D.Y. Aharon, A. Jacobi, E. Cohen, J. Tzur, M. Qadan, COVID-19, government measures and hospitality industry performance, PloS One. 16 (2021) e0255819. doi: 10.1371/journal.pone.0255819. [15] C.D. García-Gómez, D. Ender, J.M. Díez-Esteban, Y. Bilan, The impact of COVID-19 outbreak on hotels' value compared to previous diseases: the role of ALFO strategy, Heliyon. 7 (2021) e07836. doi: 10.1016/j.heliyon.2021.e07836.
16 [16] A. Ari, J.-M. Atsebi, M. Domenech, Output Losses in Europe During COVID-19: What Role for Policies?, SSRN. (2022). https://ssrn.com/abstract=4171836 (accessed January 17, 2023). [17] J. Kenny, C.S. Dutt, The long-term impacts of hotel's strategic responses to COVID-19: The case of Dubai, Tourism and Hospitality Research. 22 (2022) 71–85. doi: 10.1177/14673584211034525. [18] J. Abbas, R. Mubeen, P.T. Iorember, S. Raza, G. Mamirkulova, Exploring the impact of COVID- 19 on tourism: transformational potential and implications for a sustainable recovery of the travel and leisure industry, Current Research in Behavioral Sciences. 2 (2021) 100033. doi: 10.1016/j.crbeha.2021.100033. [19] N. Collins-Kreiner, Y. Ram, National tourism strategies during the Covid-19 pandemic, Annals of Tourism Research. (2020). doi: 10.1016/j.annals.2020.103076. [20] R. Crespí-Cladera, A. Martín-Oliver, B. Pascual-Fuster, Financial distress in the hospitality industry during the Covid-19 disaster, Tourism Management. 85 (2021) 104301. doi: 10.1016/j.tourman.2021.104301. [21] J.M. Sanabria-Díaz, T. Aguiar-Quintana, Y. Araujo-Cabrera, Public strategies to rescue the hospitality industry following the impact of COVID-19: A case study of the European Union, International Journal of Hospitality Management. 97 (2021) 102988. doi: 10.1016/j.ijhm.2021.102988. [22] B. Gopalakrishnan, R. Peters, D. Vanzetti, COVID-19 and Tourism Assessing the Economic Consequences: United Nations Conference on Trade and Development, in: Proceedings, 2020: pp. 4–27. [23] M. Niestadt, COVID-19 and the tourism sector, EPRS: European Parliamentary Research Service, 2020. [24] A. Sharma, J.L. Nicolau, An open market valuation of the effects of COVID-19 on the travel and tourism industry, Annals of Tourism Research. 83 (2020) 102990. doi: 10.1016/j.annals.2020.102990. [25] V. Krishnan, R. Mann, N. Seitzman, N. Wittkamp, Hospitality and COVID-19: How long until 'no vacancy' for US hotels?, Travel, Logistics & Infrastructure. (2020). [26] D. Guégan, B.K. Hassani, Risk measurement: From Quantitative Measures to Management Decisions, Springer International Publishing, Cham, Switzerland, 2019. [27] D. Vose, Risk analysis: a quantitative guide, John Wiley & Sons, Chichester, West Sussex, England, 2008. [28] A. Damodaran, Strategic risk taking: A framework for risk management, Pearson Education, Inc. - Prentice Hall, Upper Saddle River, New Jersey, USA, 2007. [29] B.W. Nocco, R.M. Stulz, Enterprise Risk Management: Theory and Practice, Journal of Applied Corporate Finance. 34 (2022) 81–94. doi: 10.1111/jacf.12490. [30] K.A. Froot, D.S. Scharfstein, J.C. Stein, Risk management: Coordinating corporate investment and financing policies, The Journal of Finance. 48 (1993) 1629–1658. doi: 10.1111/j.1540- 6261.1993.tb05123.x.

Ouriginal by Turnitin

17 [31] F. Knight, Risk, Uncertainty and Profit, University of Chicago Press, Chicago, USA, 1921. [32] A.Y. Rogachev, Enterprise risk management in a pharmaceutical company, Risk Management. 10 (2008) 76–84. doi: 10.1057/palgrave.rm.8250037. [33] G.A. Holton, Defining risk, Financial Analysts Journal. 60 (2004) 19–25. doi: 10.2469/faj.v60.n6.2669. [34] C. Smithson, B.J. Simkins, Does risk management add value? A survey of the evidence, Journal of Applied Corporate Finance. 17 (2005) 8–17. doi: 10.1111/j.1745-6622.2005.00042.x. [35] R.M. Stulz, Risk Management and Derivatives, Southwestern Publishing Co., Cincinnati, Ohio, USA, 2003. [36] S.W. Rawls III, C.W. Smithson, Strategic risk management, Journal of Applied Corporate Finance. 2 (1990) 6–18. doi: 10.1111/j.1745-6622.1990.tb00183.x. [37] C. Van Horne James, Financial Management and Policy, 12th ed., Prentice-Hall, Upper Saddle River (NJ), USA, 2002. [38] Z.Z. Zantout, The Sustainable Growth Rate of a Firm: A Financial and Strategic Management Model, Drexel University, 1990. [39] R.C. Higgins, How much growth can a firm afford?, Financial Management. 6 (1977) 7–16. doi: 10.2307/3665251. [40] R.A. Brealey, S.C. Myers, A. Franklin, Principles of Corporate Finance, 13th ed., McGraw-Hill, New York (NY), USA, 2020. [41] A. Ferrando, M.-T. Marchica, R. Mura, Financial flexibility and investment ability across the Euro area and the UK, European Financial Management. 23 (2017) 87–126. doi: 10.1111/eufm.12091. [42] Ö. Arslan-Ayaydin, C. Florackis, A. Ozkan, Financial flexibility, corporate investment and performance: evidence from financial crises, Review of Quantitative Finance and Accounting. 42 (2014) 211–250. doi: 10.1007/s11156-012-0340-x. [43] A. Gamba, A. Triantis, The value of financial flexibility, The Journal of Finance. 63 (2008) 2263– 2296. doi: 10.1111/j.1540-6261.2008.01397.x. [44] E. Lee, R. Powell, Excess cash holdings and shareholder value, Accounting & Finance. 51 (2011) 549–574. doi: 10.1111/j.1467-629X.2010.00359.x. [45] M. Simutin, Excess cash and stock returns, Financial Management. 39 (2010) 1197–1222. doi: 10.1111/j.1755-053X.2010.01109.x. [46] T.W. Bates, K.M. Kahle, R.M. Stulz, Why do US firms hold so much more cash than they used to?, The Journal of Finance. 64 (2009) 1985–2021. doi: 10.1111/j.1540-6261.2009.01492.x. [47] T. Opler, L. Pinkowitz, R. Stulz, R. Williamson, The determinants and implications of corporate cash holdings, Journal of Financial Economics. 52 (1999) 3–46. doi: 10.1016/S0304- 405X(99)00003-3. [48] M.-T. Marchica, R. Mura, Financial flexibility, investment ability, and firm value: evidence from firms with spare debt capacity, Financial Management. 39 (2010) 1339–1365. doi: 10.1111/j.1755- 053X.2010.01115.x.
18 [49] M.J. Barclay, C.W. Smith Jr, E. Morellec, On the debt capacity of growth options, The Journal of Business. 79 (2006) 37–60. doi: 10.1086/497404. [50] J.C. Stein, S.E. Usher, D. LaGattuta, J. Youngen, A comparables approach to measuring cashflow- at-risk for non-financial firms, Journal of Applied Corporate Finance. 13 (2001) 100–109. doi: 10.1111/j.1745-6622.2001.tb00430.x. [51] B. Stoyanov, H.W. Wieczorrek, A. Antonov, Liquidity management using Cash Flow at Risk, in: 4th International IEEE Conference Intelligent Systems, IEEE, 2008: pp. 20–8. doi: 10.1109/IS.2008.4670466. [52] H. Jankensgård, Cash-flow-at-risk and debt capacity, SSRN. (2008). [53] P. Jorion, The new benchmark for managing financial risk: Value at risk, McGraw Hill, New York, NY, USA, 2001. [54] C. Oral, G. CenkAkkaya, Cash flow at risk: a tool for financial planning, Procedia Economics and Finance. 23 (2015) 262–266. doi: 10.1016/S2212-5671(15)00358-5. [55] N. Andrén, H. Jankensgård, L. Oxelheim, Exposure-Based Cash-Flow-at-Risk: An Alternative to VaR for Industrial Companies, Journal of Applied Corporate Finance. 17 (2005) 76–86. doi: 10.1111/j.1745-6622.2005.00046.x. [56] C. Alexander, Market risk analysis, Quantitative methods in finance, John Wiley & Sons, Chichester, West Sussex, England, 2008. [57] S.A.A. Sabour, R. Poulin, Valuing real capital investments using the least-squares Monte Carlo method, The Engineering Economist. 51 (2006) 141– 160. https://doi.org/10.1080/00137910600705210. [58] M. Sauvageau, M. Kumral, Cash flow at risk valuation of mining project using Monte Carlo simulations with stochastic processes calibrated on historical data, The Engineering Economist. 63 (2018) 171–187. https://doi.org/10.1080/0013791X.2017.1413150. [59] M.V. Kok, E. Kaya, S. Akin, Monte Carlo simulation of oil fields, Energy Sources. Part B1 (2006) 207–211. https://doi.org/10.1080/15567240500400770. [60] D. Vose, Risk analysis: a quantitative guide, 3rd ed., John Wiley & Sons, Chichester, West Sussex, England, 2008. [61] G.W. Emery, K.O. Cogger, The measurement of liquidity, Journal of Accounting Research. 20 (1982) 290–303. doi: 10.2307/2490741. [62] M.T. Leary, M.R. Roberts, The pecking order, debt capacity, and information asymmetry, Journal of Financial Economics. 95 (2010) 332–355. doi: 10.1016/j.jfineco.2009.10.009. [63] H.A. Patrinos, You Can’t Manage What You Don’t Measure, (2014). https://blogs.worldbank.org/education/you-can-t-manage-what-you-don-t-measure (accessed January 17, 2023). [64] International Monetary Fund, Fiscal Monitor: A Fair Shot, IMF, Washington (DC), USA, 2021.

Ouriginal by Turnitin

19 Table 1. | Hotel population and sample The table reports the population of hotels and hotel-apartments from 2019 to 2021 (a), and the sample's distribution in 2019 (b). Hotel enterprises are legal, fiscal established entities. Each hotel enterprise may own more than one hotel and hotel-apartment unit. Portugal mainland industry a Total Percent Hotels 2019 1,286 91.3 2020 1,098 91.7 2021 1,260 91.8 Hotel-Apartments 2019 122 8.7 2020 100 8.3 2021 113 8.2 Total 2019 1,408 100.0 2020 1,198 100.0 2021 1,373 100.0 Sample (hotels and hotel-apartments) b Hotel enterprises 972 Hotel and hotel-apartment units 1,057 Table 2. | Interest coverage ratios, synthetic credit ratings, and debt spreads This table presents data on the interest coverage ratios and the debt spreads associated with the synthetic credit ratings collected from Prof. Aswath Damodaran's website (http://pages.stern.nyu.edu/~adamodar/New_Home_Page/dataarchived.html). The table also reports the sample's average interest coverage ratio, synthetic credit ratings, and debt spreads for the: Base Case (see [1]); the interest coverage ratios, synthetic credit ratings, and debt spreads are estimated under the assumption of Covid absence, and stability relating with the base case (see [2] and [4]); and the real aggregate values for the years 2020 r and 2021 r , respectively (see [3] and [5]). Damodaran's interest coverage ratios, synthetic credit ratings and debt spreads Interest coverage ratio Synthetic Rating 2019 spread (percent) 2020 spread (percent) 2021 spread (percent) -100000 0.199 D2/D 19.4 15.1 17.4 0.2 0.650 C2/C 14.5 11.0 13.1 0.65 0.800 Ca2/CC 11.1 8.6 10.0 0.8 1.250 Caa/CCC 9.0 8.2 9.5 1.25 1.500 B3/B- 6.6 5.2 6.0 1.5 1.750 B2/B 5.4 4.2 4.9 1.75 2.000 B1/B+ 4.5 3.5 4.0 2 2.250 Ba2/BB 3.6 2.4 2.8 2.25 2.500 Ba1/BB+ 2.5 2.0 2.3 2.5 3.000 Baa2/BBB 2.0 1.6 1.7 3 4.250 A3/A- 1.6 1.2 1.3 4.25 5.500 A2/A 1.4 1.1 1.2 5.5 6.500 A1/A+ 1.3 1.0 1.1 6.5 8.500 Aa2/AA 1.0 0.8 0.9 8.5 10000000 Aaa/AAA 0.8 0.7 0.7 [1] Base Case [2] projection(p) [3] real(r) [4] projection(p) [5] real(r) Average interest coverage ratio of the sample 7.6930 7.6930 -8.8915 7.6930 0.2795 Synthetic credit rating of the sample Aa2/AA Aa2/AA D2/D Aa2/AA C2/C Debt spread (percent) of the sample 1.0 0.8 15.1 0.9 13.1

20 Table 3. | Operating revenue sustainable growth rate estimation The table reports the estimates of the operating revenue sustainable growth rate using a steady-state version SGM (see Appendix I) under the following assumptions: the values of the variables used were taken directly from the databases, without intermediate estimations; Other current liabilities 2019 = Current liabilities 2019 – Payables 2019 ; Retention rate 2019 = 1- Dividend paid out 2019 / Net income 2019 . The specification of all variables can be accessed at: https://help.bvdinfo.com/mergedProjects/64_en/Home.htm. Operating revenue S 2019 Cash & equivalents / Operating revenue C&E / S 0.17 Receivables / Operating revenue RCV / S 0.05 Inventory / Operating revenue INV / S 0.06 Other current assets / Operating revenue OCA / S 0.44 Fixed assets / Operating revenue FAS / S 2.26 Payables / Operating revenue PAY / S 0.08 Other current liabilities / Operating revenue OCL / S 0.37 Net income /Operating revenue NIC / S 0.12 Retention rate r 1.55 Debt / Equity D / E 0.50 g 12.0% Table 4. | Pandemic impacts on income statement, balance sheet, and cash flow statement variables (2020-2021) (unit: 10 3 euros) The table reports the estimates on the economic and financial impact (income statement, balance sheet, and cash flow statement variables) that the pandemic had on the Portuguese hotel sector, presenting the Base Case as the year 2019 (see [1]), the deterministic estimates for the period 2020+2021 (see [2]), the real aggregate values for the same period (see [3]), the impact of the Covid-19 pandemic on the output variables measured by the difference between the 2020-2021 real values and the aggregate deterministic estimates (see [4]), and under the following assumptions: the values of the variables used were taken directly from the databases, without intermediate estimations; Net interest expense t = (Net interest expense t-1 / Non-current liabilities t- 1 * Funding needs t) + Net interest expense t-1 ; Funding needs t = Total assets t – Equity t – Non-current liabilities t – Current liabilities t ; Paid out dividend t = Net income t - Δ (Equity t – Equity t-1) under the assumption that the issuance and repurchase of shares are equivalent to each other. The specification of all variables can be accessed at: https://help.bvdinfo.com/mergedProjects/64_en/Home.htm. Panel A | Income statement [1] Base Case [2] 2020 p +2021 p [3] 2021 r +2021 r [4] (2020 r +2021 r)-(2020 p +2021 p) Operating revenue 3,492,010 8,288,356 2,970,967 -5,317,389 Cost of sales 389,307 924,027 331,730 -592,297 Operating costs 2,222,072 5,274,133 2,570,761 -2,703,372 Depreciation 311,560 739,494 570,707 -168,787 EBIT 569,071 1,350,702 -502,231 -1,852,933 Net interest expense 73,972 243,408 118,358 -125,051 P/L before tax 495,099 1,107,294 -620,588 -1,727,882 Income taxes 88,017 196,851 -42,799 -239,650 Net income 407,082 910,443 -547,332 -1,457,775 Paid out dividends -224,260 -501,561 522,013 1,023,574 Panel B | Balance sheet [1] Base Case [2] 2020 p +2021 p [3] 2021 r +2021 r [4] (2020 r +2021 r)-(2020 p +2021 p) Fixed assets 7,898,010 18,746,087 13,941,042 -4,805,045 Current assets 1,914,164 4,543,307 2,189,331 -2,353,976 TOTAL ASSETS 9,812,174 24,723,395 17,098,106 -7,625,289 Equity 8,693,769 11,445,230 7,622,391 -3,822,839 Liabilities 5,139,679 13,278,165 9,475,715 -3,802,450 Non-current liabilities 3,554,090 10,003,635 6,980,009 -3,023,625 Current liabilities 1,585,589 3,274,531 2,495,706 -778,825 TOTAL EQUITY + LIABILITIES 9,812,174 24,723,395 17,098,106 -7,625,289 Panel C | Cash flow statement [1] Base Case [2] 2020 p +2021 p [3] 2021 r +2021 r [4] (2020 r +2021 r)-(2020 p +2021 p) Operating Cash Flow 718,642 1,649,937 23,375 -1,626,562

21 Δ Working capital 620,421 332,467 -555,801 -888,268 Net Operating Cash Flow 98,221 1,317,470 579,176 -738,294 Table 5. | Pandemic impacts (2020-2021) This table presents: the Base Case as the year 2019 (see [1]); the real aggregate values for the period 2020+2021(see [2]); the deterministic estimates for the same period (see [3]); the impact of the Covid-19 pandemic on the output variables measured by the difference between the 2020-2021 the real values and aggregate deterministic estimates (see [4]); and the percent of estimates, calculated as [4]/[3]. Output variables [1] Base case (10 3 euros) [2] 2021+2021 r (10 3 euros) [3] 2020+2021 p (10 3 euros) [4] Real – Estimate (10 3 euros) [5] Percent of estimates Operating revenues 3,492,010 2,970,967 8,288,356 -5,317,389 -64.2 Net Total Assets 9,812,174 17,098,106 24,723,395 -7,625,289 -30.8 Net Total Debt 2,949,924 6,012,276 8,569,634 -2,557,358 -29.8 Net Income 407,082 -547,332 910,443 -1,457,775 -160.1 Operating Cash Flow 718,642 579,176 1,317,470 -738,294 -56.0 Financial Slack -127,236 1,529,487 8,078,267 - 6,548,780 -81.1 Debt capacity 3,308,615 8,357,450 17,801,258 - 9,443,808 -53.1 Excess cash holdings 118,239 152,047 280, 644 -128,597 -45.8 Table 6. | Impacts of the pandemic per hotel unit in 2020 and 2021 This table presents: the impact per hotel unit of the Covid-19 pandemic on the output variables, calculated as the difference between the real value and the deterministic estimate in 2020 (see [1]), and 2021 (see [2]); and the variation of those impacts in 10 3 euros (see [3]) and percentage (see [4]). The number of hotel units of the sample in 2020 and 2021, respectively, 899 and 1,030, were estimated from the real sample size in 2019 (1,057 hotel units) in proportion to the real total hotel units in Portugal mainland reported by INE (1,408 in 2019, 1,198 in 2020, and 1,373 in 2021). (INE, 2021). Output variables [1] 2020 Impact per hotel unit: [Real – deterministic estimates]/No. hotel units in 2020 (10 3 euros) [2] 2021 Impact per hotel unit: [Real – deterministic estimates]/No. hotel units in 2021 (10 3 euros) [3] Variation per hotel unit from 2020 to 2021 (10 3 euros): [2]-[1] [4] Percent variation per hotel unit from 2020 to 2021 Operating revenues -2,985 -2,557 429 -14.4 Net Total Assets -1,984 -2,933 -949 47.8 Net Total Debt -925 -1,675 -750 81.1 Net Income -1,055 -494 561 -53.2 Operating Cash Flow -925 -449 476 -51.5 Financial Slack -1,338 -5,190 -3,853 288.0 Debt capacity -2,472 -7,011 -4,538 183.6 Excess cash holdings -69 -64 5 -7.0 Table 7. | Pandemic impacts – simulation results In this table, impacts are calculated as the difference between the real values and the Monte Carlos simulation estimates (impacts measured in 10 3 euros), and the percentage of these estimates (ratio between the impact and the real value). The precision control settings were activated in Crystal Ball and set to ensure the simulation trials would stop when the standard 95 percent confidence level was reached. Simulation experiments Mean of means 1 2 3 4 5 6 7 8 9 10 Trials 50 50 50 50 50 50 50 50 50 50 Operating revenues Mean 8,007,468 8,060,278 7,998,740 8,044,370 8,034,102 8,099,004 7,947,705 8,134,236 8,052,962 8,034,122 8,041,299 Standard deviation 450,331 365,999 376,435 307,269 335,789 379,379 446,442 416,617 381,428 422,401 388,209 Impact (10 3 euros) 9,090,638 9,037,829 9,099,366 9,053,737 9,064,004 8,999,102 9,150,401 8,963,870 9,045,144 9,063,984 9,056,808 Impact (percentage) 113.5 112.1 113.8 112.6 112.8 111.1 115.1 110.2 112.3 112.8 112.7 Net total assets Mean 23,885,532 24,043,058 23,859,498 23,995,606 23,964,978 24,158,574 23,707,264 24,263,668 24,021,237 23,965,038 23,986,445 Standard deviation 1,343,295 1,091,740 1,122,870 916,556 1,001,628 1,131,653 1,331,696 1,242,729 1,137,765 1,259,984 1,157,991 Impact (10 3 euros) -6,787,426 -6,944,952 -6,761,392 -6,897,500 -6,866,872 -7,060,468 -6,609,158 -7,165,562 -6,923,131 -6,866,932 -6,888,339

Ouriginal by Turnitin

22 Impact (percentage) -28.4 -28.9 -28.3 -28.7 -28.7 -29.2 -27.9 -29.5 -28.8 -28.7 -28.7 Net total debt Mean 7,863,357 7,996,383 7,841,280 7,956,239 7,930,379 8,094,007 7,712,722 8,182,854 7,977,961 7,930,506 7,948,569 Standard deviation 1,134,950 956,880 948,802 774,494 846,357 956,244 1,125,151 1,050,063 961,319 1,064,609 981,887 Impact (10 3 euros) 9,234,749 9,101,723 9,256,826 9,141,867 9,167,727 9,004,099 9,385,384 8,915,252 9,120,145 9,167,600 9,149,537 Impact (percentage) 117.4 113.8 118.1 114.9 115.6 111.2 121.7 109.0 114.3 115.6 115.2 Net income Mean 879,005 884,727 878,063 883,004 881,892 888,925 872,531 892,742 883,933 881,894 882,672 Standard deviation 48,800 39,658 40,782 33,287 36,378 41,103 48,376 45,147 41,330 45,771 42,063 Impact (10 3 euros) -299,829 -305,551 -298,887 -303,828 -302,716 -309,749 -293,355 -313,566 -304,757 -302,718 -303,495 Impact (percentage) -34.1 -34.5 -34.0 -34.4 -34.3 -34.9 -33.6 -35.1 -34.5 -34.3 -34.4 Operating cash flow Mean 1,332,897 1,329,954 1,333,629 1,331,042 1,331,599 1,327,586 1,336,479 1,325,352 1,330,352 1,331,383 1,331,027 Standard deviation 26,720 21,900 22,545 18,460 20,115 22,787 26,482 24,946 22,676 25,188 23,182 Impact (10 3 euros) 44,543 47,486 43,811 46,399 45,841 49,854 40,961 52,088 47,088 46,057 46,413 Impact (percentage) 3.3 3.6 3.3 3.5 3.4 3.8 3.06% 3.9 3.5 3.5 3.5 Financial slack Mean 8,329,951 8,301,003 8,333,433 8,308,932 8,314,553 8,280,469 8,361,543 8,262,287 8,305,212 8,315,326 8,311,271 Standard deviation 240,764 194,632 199,436 162,399 177,805 200,972 238,491 238,491 203,389 225,078 208,146 Impact (10 3 euros) -2,317,675 -2,288,727 -2,321,156 -2,296,655 -2,302,277 -2,268,192 -2,349,266 -2,250,011 -2,292,935 -2,303,050 -2,298,994 Impact (percentage) -27.8 -27.6 -27.9 -27.6 -27.7 -27.4 -28.1 -27.2 -27.6 -27.7 -27.7 Debt capacity Mean 17,307,578 17,419,005 17,287,769 17,384,576 17,362,909 17,501,484 17,180,219 17,577,051 17,403,774 17,363,812 17,378,818 Standard deviation 956,880 778,808 801,754 654,855 715,285 808,068 948,816 886,185 811,029 898,332 826,001 Impact (10 3 euros) -15,930,138 - 16,041,565 - 15,910,328 - 16,007,136 - 15,985,469 - 16,124,044 - 15,802,779 - 16,199,611 - 16,026,334 -15,986,372 -16,001,378 Impact (percentage) -92.0 -92.1 -92.0 -92.1 -92.1 -92.1 -92.0 -92.2 -92.1 -92.1 -92.1 Excess cash holdings Mean 271,133 272,921 270,837 272,382 272,035 274,232 269,109 275,425 272,673 272,035 272,278 Standard deviation 15,248 12,393 12,746 10,404 11,370 12,846 15,117 14,107 12,915 14,303 13,145 Impact (10 3 euros) -818,465 -820,253 -818,169 -819,714 -819,366 -821,564 -816,441 -822,757 -820,005 -819,367 -819,610 Impact (percentage) -301.9 -300.6 -302.1 -301.0 -301.2 -299.6 -303.4 -298.7 -300.7 -301.2 -301.0 Cash flow at risk CFaR (10 3 euros) 1,289,972 1,294,724 1,296,050 1,299,094 1,299,968 1,293,503 1,291,871 1,289,014 1,294,308 1,288,348 1,293,685 Table 8. | Robustness checks on the pandemic impacts (2020-2021) This table presents: the real aggregate impact for the 2020-21 period [1]; the deterministic estimations for output variables (see [2]); the Monte Carlo (MC) estimations for output variables (see [3]); the aggregate impact for the 2020-21 period considering MC estimates in 10 3 euros [4] and percent [5]; the differences between the pandemic deterministic and MC impacts as a percent of real [6]; deviation of the two estimates as a percent of the deterministic estimates [7]. Output variables [1] Real 2020-21 (10 3 euros) [2] Deterministic estimates 2020-21 (10 3 euros) [3] MC estimates 2020-21 (10 3 euros) [4] Real-MC estimates 2020-21 (10 3 euros) [5] Real-MC est. as percent of MC estimates [6] percent [7] percent [3] – [1] [4] / [3] Operating revenues 2 970 967 8 288 356 8 041 299 - 5 070 332 -63.1 1.1 -1.7 Net Total Assets 17 098 106 24 723 395 23 986 445 - 6 888 339 -28.7 2.1 -6.9 Net Total Debt 6 012 276 8 569 634 7 948 569 - 1 936 292 -24.4 5.5 -18.4 Net Income - 547 332 910 443 882 672 - 1 430 003 -162.0 -1.9 1.2 Operating Cash Flow 579 176 1 317 470 1 331 027 - 751 851 -56.5 -0.5 0.8 Financial Slack 1 529 487 8 078 267 8 180 446 - 6 650 959 -81.3 -0.2 0.3 Debt capacity 8 357 450 17 801 258 17 378 818 - 9 021 368 -51.9 1.1 -2.2 Excess cash holdings 152 047 280 644 272 278 - 120 231 -44.2 1.7 -3.6 Sources: ORBIS and SABI databases; authors' estimations.

23 Appendix I – Sustainable growth model derivation Following Van Horne (2002), Zantout (1990), and Higgins (1977), we derived a steady-state version of the sustainable growth rate model to estimate the 2020 and 2021 income statements, balance sheets, and cash flow statements for a sample of the Portuguese mainland hotel industry. The model was derived under the assumption that balances of balance sheet accounts are optimized in relation to the current level of sales and that depreciations were not an available source of funds because an application of the same amount in fixed assets is assumed to sustain their operational functionality. Variable Specification S Operating revenue C&E / S Cash & equivalents / Sales RCV / S Receivables / Sales INV / S Inventory / Sales OCA / S Other current assets / Sales FAS / S Fixed assets / Sales PAY / S Payables / Sales OCL / S Other current liabilities / Sales NIC / S Net income / Sales r Net income - dividends D / E Debt / Equity where C&E denotes cash and equivalents; S, operating revenue; RCV, receivables; INV, inventory; OCA, other current assets; FAS, net fixed assets; PAY, payables; OCL, other current liabilities; NIC, net income; D, Debt; E, Equity; r, retention rate; g denotes sales sustainable growth rate. ( ) ( ) ( ) & & 1 & 1 C E RCV INV OCA FAS NIC NIC D PAY OCL

62%	MATCHING BLOCK 3/5	SA	FELLYA (34035707) BUS308 TSA 2021 Final Exam ... (D121307140)

S S S S S r S S r S S S S S S S

S S S S E S S C E RCV INV OCA FAS NIC D PAY OCL

76%	MATCHING BLOCK 4/5	SA	FELLYA (34035707) BUS308 TSA 2021 Final Exam ... (D121307140)

S S S S S r S S S S S S S

S S S E S S C E RCV INV OCA FAS NIC D NIC S S S S S r S r S S S S S S S E é ù D + D + D + D + D = +D + +D + D + D ê ú ë û æ ö D + D + D + D + D = +D + + D + D ç ÷ è ø æ ö D + D + D + D + D = + + ç ÷ è ø 1 & 1 1 & 1 1 1 D PAY OCL S S S S E S S C E RCV INV OCA FAS NIC D PAY OCL NIC D

76%	MATCHING BLOCK 5/5	SA	FELLYA (34035707) BUS308 TSA 2021 Final Exam ... (D121307140)

S S S S S r S S S r S S S

S S S S E S S S E C E RCV INV OCA FAS PAY OCL NIC D NIC D S r r S S S S S S S S S S E S E NIC D r S S E g S æ ö + D + D + D ç ÷ è ø æ ö æ ö D + D + D + D + D - + D - D - D = + ç ÷ ç ÷ è ø è ø é ù æ ö æ ö D + + + + - - - + = + ç ÷ ç ÷ ê ú è ø è ø ë û + D = = & 1 C E RCV INV OCA FAS PAY OCL NIC D r S S S S S S S S E æ ö ç ÷ è ø é ù æ ö + + + + - - - + ç ÷ ê ú è ø ë û
24 Appendix II – Variable specification Panel A | Income statement Variable Specification Operating revenue Net Sales t + Inventory variation t , Sales Net Sales t-1 x (1 + g) Cost of sales operating revenues t x (cost of sales 2019 /operating revenues 2019) Operating costs Labor Costs t + Other Operating Costs t Depreciation Operating revenues t x (Depreciation 2019 /Operating revenues 2019) EBIT Operating Revenue - Cost of Sales - Operating Costs - Depreciation Net interest expense Interest paid t + Incremental Financial Expense t – Incremental Financial Revenue t P/L before tax EBIT - Net interest expense Income taxes P/L before tax x Income tax rate Net income P/L before tax - Income taxes Paid out dividends Net income t x [Net income 2019 – (Equity 2019 - Equity 2018)] / net income 2019 Retained earnings Net income t - Paid out dividends t Panel B | Balance sheet Variable Specification Fixed assets Fixed assets t / Sales I Current assets Inventory t + Receivables t + Other Current Assets t + Cash & Equivalents t Inventory Inventory t / Sales t Receivables Receivables t / Sales t Other current assets Other Current Assets t / Sales t Cash & equivalents Operating revenues t x Cash & equivalents 2019 / Operating revenues 2019 TOTAL ASSETS Equity Equity t-1 + Retained Earnings t Liabilities Non-current liabilities Funding needs Total Assets t – Equity t - Non-current Liabilities t - Current Liabilities t Current liabilities Payables t + Other Current Liabilities t TOTAL EQUITY + LIABILITIES Panel C | Cash flow statement Variable Specification Operating Cash Flow (gross) Net Interest Expense t + Depreciation & Amortization t Δ Working capital Δ Receivables t + Δ Inventories t - Δ Payables t Operating Cash Flow Operating Cash Flow (gross) t - Δ Working capital t Panel D | Financial slack Variable Specification Debt Capacity (Earnings Before Interest & Taxes t / Interest Coverage Ratio t) / (r F + Credit Risk Premium Excess Cash Holdings Cash & Equivalents t – Liquidity Buffer t Liquidity Buffer Defense Interval t x Daily Operating Expenses t Defense Interval Current Assets t / Daily Operating Expenses t Financial Slack Debt Capacity t – Non-Current Liabilities t + Excess Cash Holdings t

Hit and source - focused comparison, Side by Side

Submitted text As student entered the text in the submitted document.

Matching text As the text appears in the source.

1/5	SUBMITTED TEXT 13 WORDS	92% MATCHING TEXT 13 WORDS
	Peter Drucker, “If you can’t measure it, you can’t manage it” [63].	Peter Drucker famously said, “If you can't measure it, you can't manage it.”

W https://blogs.worldbank.org/education/you-can-t-manage-what-you-don-t-measure

2/5	SUBMITTED TEXT	12 WORDS	65%	MATCHING TEXT	12 WORDS

Travel & Tourism Could Surpass Pre-Pandemic Levels in 2023, (2022). https://wttc.org/news-article/portugals-travel-and-tourism-could-surpass- pre-pandemic-levels-in-2023 (

travel-and-tourism-could-surpass-pre-pandemic-levels-in-2023`,` evoq_UrlQuery`:`%3fTabId%3d82%26portugals-travel-and-tourism-could-surpass-pre-pandemic-levels-in-2023`,`

W https://wttc.org/news-article/portugals-travel-and-tourism-could-surpass-pre-pandemic-levels-in-2023

3/5	SUBMITTED TEXT	16 WORDS	62%	MATCHING TEXT	16 WORDS

S S S S S r S S r S S S S S S S

SA FELLYA (34035707) BUS308 TSA 2021 Final Exam Nov.pdf (D121307140)

4/5	SUBMITTED TEXT	13 WORDS	76%	MATCHING TEXT	13 WORDS

S S S S S r S S S S S S S

SA FELLYA (34035707) BUS308 TSA 2021 Final Exam Nov.pdf (D121307140)

5/5	SUBMITTED TEXT	13 WORDS	76%	MATCHING TEXT	13 WORDS

S S S S S r S S S r S S S

SA FELLYA (34035707) BUS308 TSA 2021 Final Exam Nov.pdf (D121307140)
