# Supplementary material for: The impact of the Covid-19 pandemic on the hotel Industry’s economic performance: Evidence from Portugal
Source: Heliyon. 2023 May 3;9(5):e15850. doi: 10.1016/j.heliyon.2023.e15850 (PMC10154056; doi:10.1016/j.heliyon.2023.e15850)
Supplement: Multimedia component 2 [file mmc2.pdf]

Viper Plagiarism Report

Portuguese-Hotel-Industry_11_noreferences.pdf **scan**
21, 2023

Overall Score

0%

0.2% A Production Model for Construction: A Theor…
https://www.mdpi.com/2075-5309/5/1/209/htm

0.1% Equity valuation Activision Blizzard
https://core.ac.uk/download/481370804.pdf

The Impact of the Covid-19 Pandemic on the Hotel Industry’s Economic Performance: Evidence from Portugal*

Mário Coutinho dos Santos,a José Maganob and Jorge Motac
a CICEE, Research Center in Economics & Business Sciences, Portugal, and Catholic University of Portugal. b CICEE, and Higher Institute of Business Sciences and Tourism (ISCET), Portugal. c CICEE, DEGEIT, University of Aveiro, and GOVCOPP, Portugal.

Abstract This paper estimates the impact of the Covid-19 pandemic on the economic and financial performance of the

Portuguese mainland hotel industry. For that purpose, we implement a novel empirical approach to gauge the impact of the pandemic during the 2020-2021 period in terms of the industry's aggregated operating revenues, net total assets, net total debt, generated cash flow, and financial slack. To that end, we derive and estimate a sustainable growth model to project the 2020 and 2021 'Covid-free' aggregated financial statements of a representative Portuguese mainland hotel industry sample. The impact of the Covid pandemic is measured by the difference between the 'Covid-free' financial statements and the historical data drawn from the Orbis and Sabi databases. An MC simulation with bootstrapping indicates that the deviations of the deterministic from the stochastic estimates for major indicators vary between 0.5 and 5.5 percent. The deterministic operating cash flow estimate lies within plus or minus two standard deviations from the mean interval of the operating cash flow distribution. Based on this distribution, we estimate the downside risk, measured by cash flow at risk, at 1,294 million euros. Overall findings shed some light on the economic and financial repercussions of extreme events such as the Covid-19 pandemic, providing us with a better understanding of how to design public policies and business strategies to recover from such an impact.
Key words: cash-flow at risk; hotel industry; Covid-19; Monte Carlo simulation

JEL codes: L83, Z32, L25, C63

* Authors gratefully acknowledge the useful and valuable comments and suggestions from Celeste Eusébio, Fernando Tavares, Paulo Alves, and Rui Neves on an earlier draft, and research seminar participants at CICEE – Research Center in Economics & Business Sciences, Portugal (October 2022). We are thankful for helpful comments from an

anonymous editor and two anonymous reviewers. We also thank GOVCOPP at the University of Aveiro for providing access to AMADEUS/ORBIS and SABI databases, and INE (Statistics Portugal) for providing access to the “Sistema de Contas Integradas das Empresas” (SCIE) database. The authors are solely responsible for any inexactitudes, omissions, and errors. This paper is part of the research program “PTDC/EGEECO/4993/2021 - THE PORTUGUESE HOTEL INDUSTRY: PREPARING FUTURE AHEAD OF COVID-19 PANDEMIC” submitted to Fundação para a Ciência and Tecnologia (FCT Portugal), which did not receive any specific grant from funding agencies in the public, commercial, or not-for-profit sectors.

1. Introduction The outbreak of the Covid-19 pandemic (hereafter, referred to as the ‘pandemic’)
triggered unprecedented global disruptions in tourism and hospitality ecosystems and these sectors plunged into a severe economic and financial crisis. Unsurprisingly, such economic, financial and social impacts have a greater negative impact on economies like that of Portugal, which are much more dependent on the performance of the tourism sector than other countries.1

At the global hotel industry level, most have been forced to downsize their operations, resulting in significant economic imbalances. Ultimately, some of them may have been driven into financial distress or even insolvency. For example, the 11.1 percent operating revenue average annual growth rate of the Portuguese mainland hotel industry during the 2014-2019 period was abruptly interrupted by pandemic lockdown measures, travel bans, and other restrictions, triggering unprecedented

disruptions and plunging the sector into economic turmoil, with grievous social-economic externalities [1]. Notably, it led to a generalized scaledown of hotel operations, plummeted revenues, fueled workforce layoff, and exposed the ecosystem to economic and financial disarray [5-7]. Although prospects for the recovery of the tourism sector remained relatively unsteady, governmental policymakers and hotel ecosystem participants alike strove to map out alternative feasible recovery paths (and pace) for designing post-pandemic resilient and sustainable recovery strategies for the industry (e.g., [8]).

The most recent research on the topic examines either public policy aimed at mitigating the impact of COVID-19 on tourism (e.g., [9-11]), or explores particular performance dimensions of specific segments of the hotel industry, such as listed hotels (e.g., [12-15]).2 Yet, the development of blueprints to support the design of prospective strategic scenarios requires the availability of a comprehensive and quantitative assessment of the magnitude of the economic, financial, and social impacts of the Covid-19 shock. To our knowledge, no such assessment has been carried out, motivating us to perform this study.

The main research objective of this paper is to quantitatively assess the potential economic and financial shocks on the performance of the Portuguese hotel industry. Data are based on approximately 1,000 hotels in mainland Portugal in the period 2020-2021.

Besides providing evidence of the aggregated pandemic's impact on the economic and financial condition of the industry, this work also contributes to the literature by applying a novel methodological approach. First, it derivates and estimates a deterministic business model

1 According to Instituto Nacional de Estatística [1], the direct and indirect contribution of the tourism industry to GDP dropped from

11.8 percent in 2019 to 6.6 percent in 2020 and rose to 8.0 percent in 2021 (see also, [2-4]). 2 For further details on the pandemic's impacts on the tourism industry, see, e.g., [16-25].
2

for the sector, anchored on the maximum sustainable growth rate (SGM) conceptual framework to gauge the impact of the pandemic during the 2020-2021 period in terms of operating revenues, net total assets, net total debt, operating cash flow, and financial slack.3 Next, this approach combines with a robustness check that incorporates stochastic variability into the deterministic base case by applying Monte Carlo (MC) methods with bootstrapping to measure the downside risk of our hotel industry sample using the cash-flow-at-risk (CFaR) conceptual framework.

2. Conceptual background 2.1 Maximum sustainable growth framework

Tourism and hospitality industries are exposed to a wide spectrum of risks. Therefore, enterprise risk management (ERM) — risk identification, measurement, and management — is instrumental in helping firms to manage their value creation objectives, particularly in terms of mitigating financial distress and optimizing risk portfolio (e.g., [26-30]).4

At the hotel firm level, the measurement of the expected impact of downside risk factors on value creation should be a primary managerial concern (e.g., [28, 34-36]. Further, there is abundant and compelling evidence that the volatility in corporate accounting aggregates, such as net income and operating cash flows, is related to value creation (e.g., Smithson & Simkins, 2005 [34]). Under well-diversified firm ownership, risk management can be expected to be positively related to a firm's value, which could limit the expected costs of financial distress,

manage financial slack, reduce tax liability, and mitigate suboptimal resource allocation (e.g., [35]).
To estimate the economic and financial impact of the Covid-19 pandemic on the Portuguese mainland hotel industry in 2020 and 2021, we develop a novel empirical, methodological approach based on the SGM framework. SGM builds on the percentage-ofsales method's standard assumptions that the stocks of the balance sheet accounts are optimized

3 Our deterministic estimations incorporate the Covid-19 mitigating measures decided at the domestic government level, within the scope of the policies implemented at the European Union level, to support the tourism sector during the pandemic period. Measures include, but are not limited to, credit lines, flexibility in tax payment, and the deferral of the payment of social benefits (Portuguese versions are available at: https://www.bportugal.pt/sites/default/files/anexos/nota_sobre_as_prir https://www.portugal.gov.pt/pt/gc22/comunicacao/comunicado?i=governo-adota-medidas-para-apoiarempresas-e-trabalhadores; https://www.portugal.gov.pt/pt/gc22/comunicacao/noticia?i=governo-toma-medidasextraordinarias-para-responder-a-epidemia-de-covid-19; http://business.turismodeportugal.pt/pt/Gerir/covid19/Paginas/medidas de-apoio-economia.aspx; all accessed on October 5, 2022). 4 'Risk' and 'uncertainty' are often used interchangeably. However, as insightfully articulated by Frank H. Knight in 1921 [31], the two concepts have distinct meanings: risk relates to objective probabilities, whereas uncertainty relates to subjective probabilities (see also [32,33]).

in relation to the current level of sales and vary in proportion to

sales; and that depreciation and amortization are not an available source of funds because it is assumed that the same amount is applied in restoring fixed assets operational functionality (e.g., [37-39]).5

Under the maximum annual percentage increase in operating revenue — g — a firm can sustain, keeping constant at the pre-pandemic levels (2019): (i) fixed assets utilization, proxied by the net fixed assets-to-operating revenue ratio; (ii) after-tax operating revenue profitability, measured by the net income-to-operating revenue ratio; (iii) capital structure gauged by the debt-to-equity ratio; and (iv) the retention rate of earnings, measured by the complement of the dividend payout ratio, and without resorting to incremental external funding.

Financial slack (FS) is a readily available liquidity cushion in the form of excess cash holdings and debt capacity, which provides financing flexibility by mitigating the impact of adverse liquidity shocks, and financial distress and by moderating suboptimal allocative behavior, namely in the form of underinvestment (e.g., [13]).6 Therefore, for precautionary reasons, firms tend to accumulate liquid assets, such as cash and equivalents, as an 'insurance' against liquidity shortfalls arising in adverse states of cash flow generation and to avoid asset fire sales, raising externally costly unanticipated funding, or incurring inefficient underinvestment (e.g., [40-43]).

Under this framework, firms with higher asset systematic riskiness and costlier access to external capital markets tend to carry larger cash holdings on their balance sheets. We measure excess cash holdings as the difference between "Cash & Equivalents" and the "Liquidity Buffer" balances (e.g., [44-47]).7

Conceptually, debt capacity is the incremental borrowing required to sustain the capital market's perception of a firm's current aggregate asset systematic riskiness. Or restated, the

maximum amount that could optimally be borrowed at the current risk-adjusted marginal cost of debt (e.g., [40, 48, 49]).

2.2 Cash-flow-at-risk Another valuable tool to assess non-financial firms' downside risk is cash flow at risk (CFaR). CFaR is a composite measure of the maximum decrease in expected cash flows associated with the uncertainty of risk factors, given a pre-defined confidence level, for a given

5 See Appendix I for the derivation of a steady state pre-pandemic version of the sustainable growth rate model, derived to estimate the financial income statements for 2020 and 2021. 6 Henceforth, we use 'financial flexibility' and 'financial slack' interchangeably. 7 See Panel D of Appendix II. It should be noted that cash holding balances are firm-specific and determined by firm characteristics, such as size, cash flow generation profile, growth opportunities set, and firm positioning in its life cycle.

period, which Stein et al. [50] define "as the probability distribution of a company's operating cashflows over some horizon in the future, based on information available today". Taking a prespecified timeframe and statistical confidence level, the CFaR approach to downside risk measurement estimates the maximum shortfall of cash a firm is willing to accept and, therefore, its overall liquidity risk over a given period (e.g., [28, 50-52].8 Moreover, since all risk exposures can be aggregated into a single metric, CFaR provides quantitative information, at least accurate on average, helping to guide managerial decision-making (e.g., [35, 54, 55]). Indeed, 'it is the "lower tail" of the cash flow distribution that can have costly consequences, such as insufficient funds to carry out the company's investment program or even bankruptcy' [55]; CFaR provides a measure of

such lower tail effects, which we estimate by bootstrapping the Portuguese mainland hotel industry model for robustness-checking purposes, following Alexander [56].

It should be emphasized that the data panel used in the deterministic methodological approach features an inherent statistical significance limitation, which inhibits inferential testing. The estimation of CFaR with Monte Carlo (MC) simulation has been addressed in the literature, namely, through examples that suggest that this numerical tool is effective for solving problems in finance that involve closed-form analytical solutions that are too complex or impossible to determine (e.g., [57-58]). Besides the benefit of efficiently dealing with complexity, another advantage of MC is its inherent randomness, which is essential for simulating real-life random systems [59]. This method is, therefore, an obvious choice for tackling the CFar estimation as a robust check in our study's approach. Hence, we use MC bootstrapping computational methods to perform the number of trials necessary in each simulation experiment to generate a numerical approximation to the true distribution of the output variable at the standard 95 percent confidence level.9

3. Methods 3.1 Research design

This paper examines the impact of the Covid-19 pandemic period during the 2020 and 2021 sampling periods on the Portuguese mainland hotel industry. Specifically, we estimate

8 Conceptually, CFaR is a metric similar to value-at-risk (VaR), which "summarizes the worst loss over a target horizon that will not be exceeded with a given level of confidence" [53]. Specifically, VaR describes the quantile of the projected distribution of gains and losses over a target horizon. Specifying c as the selected confidence level, VaR corresponds to the 1-c lower tail level. Unlike CFaR, VaR does not include the time value of money because the short time horizon does not require

periodic cashflows to be discounted [27] . 9 See [60] for a comprehensive description of the use of the MC approach in risk analysis.

the pandemic effect in terms of total net assets, total net debt, operating cash flow, and financial flexibility, for a representative sample of Portugal's mainland hotels (hereafter, referred to as the 'sample').

The empirical implementation strategy is designed in five steps. First, we estimate aggregate balance sheets, income, and operating cash flow statements for the 2014-2021 sampling period, drawing data from the Orbis/Sabi databases. Second, we derive a steady-state version of SGM and estimate the operating revenue sustainable growth rate to forecast the sample's financial statements for 2020 and 2021.10 Regarding the third step, we project 2020 and 2021 aggregate balance sheets, income statements and cash flow statements, which are unconditioned by the occurrence of the Covid-19 pandemic. Fourth, we measure the (deterministic) impact of the Covid-19 pandemic as the difference between the projected and observed 2020's and 2021's aggregated operating revenues, net total assets, total net debt, operating cash flow, and financial slack. Fifth, we run a Monte Carlo simulation experiment to check for the robustness of the deterministic 2019 base case in terms of the output variables required to compute the CFaR.

3.2 Data Economic, financial, and operating data for this research were drawn from INE

(Statistics Portugal), Sabi, and Orbis, covering the 2010-2021 sampling period. However, to minimize the spillovers of the financial assistance program signed between Portugal and the International Monetary Fund, the European Union, and the

European Central Bank, encompassing the application of a three-year economic adjustment program (2011-mid-2014), we restricted the sampling period to 2014-2019.

Results of summary statistics and parametric tests for equality of means document that the variables used in the deterministic model do not exhibit, at the standard confidence levels, statistically significant differences in means between the sampling subperiods of 2010-2019 and 2014-2019. These results support the consistency of using the 2014-2019 subsampling period for this empirical analysis and segmenting hotels by star category.11

To be included in the sample, a hotel must comply with the following criteria: (i) be included in the National Register of Tourism Enterprises (RNET) database, with an assigned fiscal number; (ii) be established and operating on Portugal's mainland (iii) be a star classified

10 See, Appendix I for the derivation of the sustainable growth rate model. 11 Test results are available from the corresponding author upon request.

hotel or hotel-apartment; (iv) be active for the entire sampling period.12 The population of hotels and hotel-apartments was drawn from the INE database for the 2019-2021 period (Table 1).

<INSERT TABLE 1 HERE>

A search in the RNET database yielded a sample of 1,282 hotel units with assigned fiscal numbers and star classifications that met the above-mentioned criteria. Finally, we identified 972 entities in Orbis/Sabi databases that matched that set and reported complete data for the 2019-2021 period, which corresponded to 1,057 hotel and hotel-apartment units. For this

sample, we extracted economic, financial, and operating data at the hotel business firm level (Table 1).13

Data on income statement items, such as labor costs, other operating costs, financial revenues, and financial expenses, were drawn from the Sabi database. In addition, data on the interest coverage ratios and the debt spreads associated with the synthetic credit ratings were collected from Prof. Aswath Damodaran's website;14 10-year government bond yields for Portugal and triple-A rating countries were collected from the European Central Bank's Statistical Data Warehouse.

3.3 SGM modeling and estimation Aggregate balance sheets, income, and cash flow statements were estimated using the variable specified in Appendix II. Financial slack is modeled as the sum of excess cash holdings (ECH) and debt capacity (DC). ECH is measured as the difference between the book value of cash and equivalents, and the minimum cash balance requirements (e.g., [45]).15 We use the defensive interval ratio (DIR) to estimate the short-term liquidity needs in terms of the number of days a hotel could operate resorting only to its current assets (e.g., [61]):

12 The National Register of Tourism Enterprises (RNET) provides a database on tourism firms with a valid license, including data on, e.g., firm denomination, type of tourism unit, capacity, location, and age: http://business.turismodeportugal.pt/pt/Planear_Iniciar/Licenciamento_Turisticos/Paginas/rnet.aspx. 13 Data on the sampled firms for the year 2021 were drawn from the Orbis/Sabi databases on September 30, 2022, when only 915 of the 972 sample firms had disclosed their data to the database. Therefore, we estimate 2021 financial statements as a direct proportion, using 915/972 as a constant of proportionality. 14 Data available at: http://pages.stern.nyu.edu/~adamodar/New_Home_Page/dataarchive

15The “Liquidity Buffer” (the minimum cash balance requirement) is specified as the product of the 2019 defensive interval ratio by the daily operational expenses.
7

DIR =

Current Assetst

Daily Operating Expensest

(1)

where current assets include cash, marketable securities, and net receivables; and daily

operational expenses are measured by the sum of the cost of sales, operating costs, and net

interest rate divided by 360.

We define DC as the maximum amount of borrowing lenders are willing to extend to

an investment-grade rated firm based on its interest coverage ratio and the debt spread

associated with its rating notation (see, e.g., [62]). Hotel’s DC is specified as:

Earnings Before Interestst & Taxest

Debt Capacity =

Interest Coverage Ratiot

(2)

rF + spread

where rF denotes the rate of return on a riskless asset; and spread, the debt’s default risk

premium.

We estimate hotel synthetic rating notations, interest coverage ratios, and the debt

default spreads associated with them using Aswath Damodaran’s website [28] (Table 2). The

model uses the operating income (EBIT) and the net interest expense as inputs to estimate the

interest coverage ratio, which is extensively used by Standard and Poor's and Moody's, two

leading international rating agencies.

<INSERT TABLE 2 HERE>

3.4 MC approach We use MC computational numerical methods with bootstrapping to produce forecasts
of the 2020 and 2021 aggregated operating revenues, net total

assets, net total debt, operating cash flow, financial slack, debt capacity, and excess cash holdings to check for the robustness of the deterministic model. A single independent variable — operating revenues sustainable growth rate — is randomized, specified as a triangular distribution, and heuristically calibrated as follows: minimum = 0 percent; likeliest value = 12 percent; and maximum = 17 percent.16 The MC simulation follows a bootstrap multiple-simulation approach, repeatedly simulating the model and then creating a distribution of the statistics from each simulation, as depicted in Figure 1.

16 The Monte Carlo simulation was carried out using the “Oracle Crystal Ball” add-in to MS-Excel, release 11.1.2.4.900 (64-bits).

Figure 1 – Bootstrap multiple-simulation method
Source: Crystal Ball User Guide.
4. Results 4.1 SGM estimation
Data in Table 1 document that during the Covid-19 shock, the hotel population diminished relatively to 2019. However, it only partially recovered in 2021 without achieving pre-pandemic levels. We estimate the 2019 ‘Covid-19-free’ operating revenue sustainable growth rate, using the steady-state SGM model derived in Appendix I, at 12.0 percent (see Table 3). We used this growth rate to project pro forma aggregate balance sheets, income, and cash flow statements for the 2020 and 2021 periods (see Appendix II for the specification of the variables).
<INSERT TABLE 3 HERE> Panels A, B, and C of Table 4 present the estimation of the sample’s aggregate financial statements for the 2020 and 2021 periods, based on the 2019 period’s SGR estimate unconditioned by the Covid-19 outbreak. The table presents the 2019 base case (column [1]); the

deterministic estimations for 2020 and 2021 aggregate pro forma balance sheet, income, and cash flow statements (columns [2] and [3]); the 2020 and 2021 real aggregate balance sheet, income, and cash flow statements (columns [4] and [5]); and the estimates of the impact of the Covid-19 pandemic on the output variables measured by the differences between the 2020-2020 and 2021-2021 real aggregate values and the deterministic estimates (columns [6] and [7]).
<INSERT TABLE 4 HERE>

The deterministically estimated aggregate impacts inflicted on our Portuguese mainland hotel industry sample over the 2020-2021 pandemic period are presented in Table 5:
<INSERT TABLE 5 HERE> The estimated economic impacts measured by the aggregated operating revenues, net income, and operating cash flow are: a 64.2 percent reduction (-5,317 million euros); a 160.1 percent decrease (-1,457 million euros); and a 56.0 percent drop (-738 million euros), respectively. The financial repercussions, gauged by the variation in the non-current liabilities and the financial slack, are: a 29.9 percent increase (+2,557 million euros); and an 87.1 percent decline (-9,316 million euros), respectively. The latter impact is the compound effect of the 53.1 percent fall in debt capacity (-9,444 million euros) and the 45.8 percent reduction in the excess cash holdings (-129 million euros). Despite the reported aggregate impacts of 2020 and 2021, we must recognize that, after a severe decrease in hotel demand in 2020, some recovery was seen in 2021. Accordingly, our model presents different impact variations between the estimated and real outcomes from 2020 to 2021, of which it is worth mentioning the following (i) a 50 million euro increase in operating revenues (1.9 percent); (ii) a

1,238 million euro drop in the industry's net total assets (69.4 percent); (iii) an 894 million euro increase in the sector's non-current liabilities balance (107.4 percent); (iv) a 440 million euro increase in net income (46.4 percent); (v) a 369 million euro increase in the operating cash flow (44.4 percent); (vi) a 4,144 million euro drop in financial slack (344.5 percent); (vii) a 4,998 million euro drop in financial slack (224.9 percent); and (viii) a 4 million euro drop in financial slack (6.6 percent). To summarize, in 2021, net income and operating cash flow improved due to growing demand, whereas net total assets, net total debt, and financial slack worsened (Figure 2).

Figure 2 – The impact of the Covid-19 pandemic: 2020, 2021, and 2020-21 (unit: 103 euros).

The launch of the vaccination campaign by the end of 2020 may have, to a certain extent, lessened tourist travel restrictions, creating the conditions necessary for some recovery in hotel occupancy rates in 2021 and, consequently, in operating revenues. However, as shown in Figure 2, the difference between 2020 and 2021 operating revenues is relatively marginal. We conjecture that this might have been the effect of the phasing out in 2021 of the governmental Covid-19 mitigating measures in place.

Debt capacity and excess cash holdings are the two sources of financial flexibility required to mitigate potential liquidity shortfalls and suboptimal allocative behavior in adverse states of the world, such as during the Covid-19 outbreak (See Panel D in Appendix II for the specification of the variables). Results document that over the 2020-2021 period, the hotel industry suffered a major fall-off in financial flexibility due, first and foremost, to debt capacity contraction, which seriously curtails

potential recovery funding needs, notably in the new context of central banking's monetary tightening policy. The breakdown of financial slack indicates that borrowing capacity is its main determinant (see Table 5 and Figure 2).

To sharpen the analysis of the results, we estimate the 2020 and 2021 impacts of the output variables at the hotel unit level (see Table 6). As expected, the impacts on accounting economic aggregates, such as operating revenues, net income, and operating cash flow, were more negative in the first year of the pandemic.

<INSERT TABLE 6 HERE>

4.2 Robustness check: Monte Carlo simulation As previously described, we followed a bootstrap MC multiple-simulation approach,
repeatedly running the model. As such, ten simulation experiments were performed, each one with the number of trials required to generate a numerical approximation to the distribution of the output variables.17 Results are summarized in Table 7.18

<INSERT TABLE 7 HERE>

In each experiment, cash flow at risk was estimated as the operating cash flow for which the accumulated probability of occurrence is 5 percent, or, in other words, the likelihood of

17 The grand mean of the ten independent and identically distributed simulations is similar, under the linearity assumption, to generating ten samples from one simulation only. Nonetheless, the greater the number of simulation trials we run, the more the resulting means distribution will tend towards the true output variable distribution. 18 Detailed results are available from the authors upon reasonable request from registered Oracle Crystal Ball (64bits) licensees.

exceeding CFaR is 95 percent. The Monte Carlo simulation results are very close to those obtained with the deterministic approach described in section 4. Table 8 exhibits the robustness check estimates performed using Monte Carlo simulation methods with bootstrapping for the net total assets, net total debt, operating cash flow, and financial slack at risk, as well as the aggregate impact of the Covid-19 pandemic for the 2020-21 period. These suggest that, on average, the absolute deviations between deterministic and stochastic estimates at 95 percent confidence level are relatively minor, oscillating between 0.5 and 5.5 percent.

<INSERT TABLE 8 HERE> In all iterations of the simulation experiment, the deterministic operating cash flow, as well as the grand mean, is within plus or minus two standard deviations from the mean interval of the operating cash flow distribution (see Figure 3).

Figure 3 – Comparison of the deterministic operating cash flow (1,317,470 103 euros) with the MC cash flow estimates (103 euros) obtained in each iteration and the mean of means.

Legend: CF = operating cash flow; SD = standard deviation.

5. Discussion and Conclusions According to Peter Drucker, “If you can’t measure it, you can’t manage it” [63]. Thus,

the resilient and sustainable recovery from the Covid-19 pandemic requires the ex-ante substantiated assessment of the extent of the repercussions of the pandemic. The main research purpose of this paper is to contribute to that end. As such, we examine the economic and financial impacts on the performance of the Portuguese mainland hotel industry during the 2020-2021 Covid-19 pandemic period in terms of the industry’s aggregate operating revenues, net total assets, net total debt, generated cash flow, and financial slack, using a deterministic approach

and stochastic robustness checking. We found that, on average, over the 2020-2021 period, the Covid-19 pandemic inflicted an aggregate impact of: (i) a 64.2 percent reduction

in operating revenues (-5,317 million euros); (ii) a 30.8 percent decrease in the industry's net total assets (-7,625 million euros); (iii) a 29.8 percent increase in the sector’s indebtedness (+2,557 million euros); (iv) a 160.1 percent decrease in net income (-1,457 million euros); (v) a 56.0 percent drop in the operating cash flow (-738 million euros); (vi) an 81.1 percent decline in financial slack (-9,443 million euros). Overall, these (deterministic) findings, on the one hand, quantify the extent of the economic problem caused by the pandemic. On the other hand, they provide estimations of the economic thresholds to be overcome and the financial hurdles faced by the future sustainable recovery of the industry.

The robustness check, conducted through the MC simulation with bootstrapping, indicates that the deviations of the deterministic from the stochastic estimates are, at a 95 percent confidence interval: 1.1 percent for the operating revenues; 2.1 percent for the net total assets; 5.5 percent for the net total debt; 1.9 percent for the net income; 0.5 percent for the operating cash flow; and 0.5 percent for the financial slack.

Overall, not only the stochastic approach delivers comparable outputs for the variables at interest, but unlike the deterministic approach, only the output analysis of the simulation output can be used to understand what happens at the lower tail of the resulting cash flow distribution. In addition, the bootstrap MC approach allowed for an estimate of the uncertainty of the operating cash flow, resulting in an expected downside risk of the Portuguese mainland hotel industry over the 2020-2021

period of 1,293 million euros. In other words, such is the maximum shortfall of our industry sample's generated net cash flow during the Covid-19 period. In this framework, the MC model provides more information than the deterministic model and is a valuable tool for assessing the effects of the Covid-19 pandemic on the Portuguese mainland hotel industry.

The 2020 and 2021 impacts on accounting economic aggregates, such as operating revenues, net income, and operating cash flow, were more negative in the first year of the pandemic, as expected. We conjecture that this is due to the initial rounds of government lockdown measures, travel bans, and restrictions. However, the massive vaccination deployment, and the governmental fiscal policy measures to provide emergency countercyclical support to households and firms, may have contributed to the downscaling of the impacts of those effects in 2021 (e.g., [64]). The financial impacts, measured, for example, by the stocks of net total debt and financial flexibility, reflect, among other factors, the funding needs associated with the negative cash flow generation over the 2020-2021 period, and the effect of

the moratoriums on bank credit agreements enacted in March 2020 because of the Covid-19 health emergency.19

We can conclude that the extreme uncertainty and volatility associated with events, such as the Covid-19 pandemic, can expose business activity to extremely adverse economic and financial consequences. Our findings are consistent with the claim that that was the case in the Portuguese hotel industry.

A limitation of this study is that it focused only on hotels (ORBIS/Sabi databases do not cover very small accommodation units) and treated the sample as a whole without distinguishing

hotel size or exploring possible recovery strategies. As such, future research on this topic should develop along two axes. The first is to use a difference-in-differences approach to study whether or not hotels grouped by star classification were impacted differently by the Covid-19 pandemic. The second, building on the conceptual framework that business strategies can be conceptualized as chains of real options, is to develop a randomized valuation framework to appraise the value creation potential of the post-pandemic recovery strategies of the hotel industry.

19 See Decree of Law No. 10 J/2020 of 26 March 2020; https://www.bportugal.pt/en/comunicado/covid-19moratorium-credit-agreements-bank-customers-entered-force, accessed on October 24, 2022.

References 15

Table 1. | Hotel population and sample

The table reports the population of hotels and hotel-apartments from 2019 to 2021 (a), and the sample's distribution in 2019 (b). Hotel enterprises are legal, fiscal established entities. Each hotel enterprise may own more than one

hotel and hotel-apartment unit.

Portugal mainland industrya Hotels

2019

Total 1,286

Percent 91.3

2020

1,098

91.7

2021

1,260

91.8

Hotel-Apartments

2019

122

8.7

2020

100

8.3

2021

113

8.2

Total

2019

1,408

100.0

2020

1,198

100.0

2021

1,373

100.0

Sample (hotels and hotel-apartments)b

Hotel enterprises

972

Hotel and hotel-apartment units

1,057

Table 2. | Interest coverage ratios, synthetic credit ratings, and debt spreads

This table presents data on the interest coverage ratios and the debt spreads associated with the synthetic credit ratings collected from Prof. Aswath Damodaran's website (http://pages.stern.nyu.edu/~adamodar/New_Home_Page/dataarchive
The table also reports the sample's average interest coverage ratio, synthetic credit ratings, and debt spreads for the: Base Case (see [1]); the interest coverage ratios, synthetic credit ratings, and debt spreads are estimated under the assumption of Covid absence, and stability relating with the base case (see [2] and [4]); and the real aggregate values for the years 2020r and 2021r, respectively (see [3] and [5]).
Damodaran's interest coverage ratios, synthetic credit ratings and debt spreads

Interest coverage ratio

-100000

0.199

Synthetic Rating
D2/D

2019 spread (percent)
19.4

2020 spread (percent)
15.1

2021 spread (percent)
17.4

0.2

0.650

C2/C

14.5

11.0

13.1

0.65

0.800

Ca2/CC

11.1

8.6

10.0

0.8

1.250

Caa/CCC

9.0

1.25

1.500

B3/B-

6.6

8.2

9.5

5.2

6.0

1.5

1.750

B2/B

5.4

4.2

4.9

1.75

2.000

B1/B+

4.5

3.5

4.0

2

2.250

Ba2/BB

3.6

2.4

2.8

2.25

2.500

Ba1/BB+

2.5

2.5

3.000

Baa2/BBB

2.0

2.0

2.3

1.6

1.7

3

4.250

A3/A-

1.6

1.2

1.3

4.25

5.500

A2/A

1.4

1.1

1.2

5.5

6.500

A1/A+

1.3

1.0

1.1

6.5

8.500

Aa2/AA

1.0

8.5

10000000

Aaa/AAA

0.8

0.8

0.9

0.7

0.7

[1]

[2]

[3]

[4]

[5]

Average interest coverage ratio of the sample

Base Case projection(p)

7.6930

7.6930

real(r)
-8.8915

projection(p)
7.6930

real(r)
0.2795

Synthetic credit rating of the sample

Aa2/AA

Aa2/AA

D2/D

Aa2/AA

C2/C

Debt spread (percent) of the sample

1.0

0.8

15.1

0.9

13.1

16

Table 3. | Operating revenue sustainable growth rate estimation
The table reports the estimates of the operating revenue sustainable growth rate using a steady-state version SGM (see Appendix I) under the following assumptions: the values of the variables used were taken directly from the databases, without intermediate estimations; Other current liabilities2019 = Current liabilities2019 – Payables2019; Retention rate2019 = 1- Dividend paid out2019 / Net income2019. The specification of all variables can be accessed at:
https://help.bvdinfo.com/mergedProjects/64_en/Home.htm.

Operating revenue Cash & equivalents / Operating revenue Receivables / Operating revenue Inventory / Operating revenue Other current assets / Operating revenue
Fixed assets / Operating revenue Payables / Operating revenue Other current liabilities / Operating revenue Net income /Operating revenue Retention rate Debt / Equity

S C&E / S RCV / S INV / S OCA / S
FAS / S PAY / S OCL / S NIC / S
r D/E
g

2019 0.17 0.05 0.06 0.44
2.26 0.08 0.37 0.12 1.55 0.50 12.0%

Table 4. | Pandemic impacts on income statement, balance sheet, and cash flow statement variables (2020-2021) (unit: 103 euros)
The table reports the estimates on the economic and financial impact (income statement, balance sheet, and cash flow

statement variables) that the pandemic had on the Portuguese hotel sector, presenting the Base Case as the year 2019 (see [1]), the deterministic estimates for the period 2020+2021 (see [2]), the real aggregate values for the same period (see [3]), the impact of the Covid-19 pandemic on the output variables measured by the difference between the 2020-2021 real values and the aggregate deterministic estimates (see [4]), and under the following assumptions: the values of the variables used were taken directly from the databases, without intermediate estimations; Net interest expenset = (Net interest expenset-1 / Non-current liabilitiest1 * Funding needst) + Net interest expenset-1; Funding needst = Total assetst – Equityt – Non-current liabilitiest – Current liabilitiest; Paid out dividendt = Net incomet - Δ (Equityt – Equityt-1) under the assumption that the issuance and repurchase of shares are equivalent to each other. The specification of all variables can be accessed at: https://help.bvdinfo.com/mergedProjects/64_en/Home.htm.

Panel A | Income statement

[1]

Base Case

Operating revenue

3,492,010

Cost of sales

389,307

Operating costs

2,222,072

Depreciation

311,560

EBIT

569,071

Net interest expense

73,972

P/L before tax

495,099

Income taxes

88,017

Net income

407,082

Paid out dividends

-224,260

[2] 2020p+2021p
8,288,356 924,027 5,274,133 739,494 1,350,702 243,408
1,107,294 196,851 910,443 -501,561

[3] 2021r+2021r
2,970,967 331,730 2,570,761 570,707 -502,231 118,358
-620,588 -42,799 -547,332 522,013

[4] (2020r+2021r)-(2020p+2021p)
-5,317,389 -592,297 -2,703,372 -168,787 -1,852,933 -125,051
-1,727,882 -239,650 -1,457,775 1,023,574

Panel B | Balance sheet

[1]

Base Case

Fixed assets

7,898,010

Current assets

1,914,164

TOTAL ASSETS

9,812,174

Equity

8,693,769

Liabilities

5,139,679

Non-current liabilities

3,554,090

Current liabilities

1,585,589

TOTAL EQUITY +

9,812,174

LIABILITIES

[2] 2020p+2021p
18,746,087 4,543,307 24,723,395 11,445,230 13,278,165
10,003,635 3,274,531 24,723,395

[3] 2021r+2021r
13,941,042 2,189,331 17,098,106 7,622,391 9,475,715
6,980,009 2,495,706 17,098,106

[4] (2020r+2021r)-(2020p+2021p)
-4,805,045 -2,353,976 -7,625,289 -3,822,839 -3,802,450
-3,023,625 -778,825 -7,625,289

Panel C | Cash flow statement

[1]

Base Case

Operating Cash Flow

718,642

[2] 2020p+2021p
1,649,937

[3] 2021r+2021r
23,375

[4] (2020r+2021r)-(2020p+2021p)
-1,626,562

17

Δ Working capital Net Operating Cash Flow

620,421 98,221

332,467 1,317,470

-555,801 579,176

-888,268 -738,294

Table 5. | Pandemic impacts (2020-2021)

This table presents: the Base Case as the year 2019 (see [1]); the real aggregate values for the period 2020+2021(see [2]); the deterministic estimates for the same period (see [3]); the impact of the Covid-19 pandemic on the output variables measured by the difference between the 2020-2021 the real values and aggregate deterministic estimates (see [4]); and the percent of estimates, calculated as [4]/[3].

Output variables
Operating revenues Net Total Assets Net Total Debt Net Income Operating Cash Flow Financial Slack Debt capacity Excess cash holdings

[1] Base case (103 euros)
3,492,010 9,812,174 2,949,924 407,082 718,642 -127,236 3,308,615 118,239

[2]
2021+2021r (103 euros) 2,970,967 17,098,106 6,012,276
-547,332
579,176 1,529,487 8,357,450
152,047

[3]
2020+2021p (103 euros) 8,288,356 24,723,395 8,569,634 910,443 1,317,470 8,078,267 17,801,258
280, 644

[4] Real – Estimate
(103 euros)
-5,317,389 -7,625,289 -2,557,358 -1,457,775 -738,294 -

6,548,780 - 9,443,808 -128,597

[5] Percent of estimates
-64.2 -30.8 -29.8 -160.1 -56.0 -81.1 -53.1 -45.8

Table 6. | Impacts of the pandemic per hotel unit in 2020 and 2021

This table presents: the impact per hotel unit of the Covid-19 pandemic on the output variables, calculated as the difference between the real value and the deterministic estimate in 2020 (see [1]), and 2021 (see [2]); and the variation of those impacts in 103 euros (see [3]) and percentage (see [4]). The number of hotel units of the sample in 2020 and 2021, respectively, 899 and 1,030, were estimated from the real sample size in 2019 (1,057 hotel units) in proportion to the real total hotel units in Portugal mainland reported by INE (1,408 in 2019, 1,198 in 2020, and 1,373 in 2021). (INE, 2021).

Output variables
Operating revenues Net Total Assets Net Total Debt Net Income Operating Cash Flow Financial Slack Debt capacity Excess cash holdings

[1] 2020 Impact per hotel
unit: [Real – deterministic estimates]/No. hotel units in 2020 (103 euros)
-2,985 -1,984 -925 -1,055 -925 -1,338 -2,472
-69

[2] 2021 Impact per hotel
unit: [Real – deterministic estimates]/No. hotel units in 2021 (103

euros)
-2,557 -2,933 -1,675 -494 -449 -5,190 -7,011
-64

[3] Variation per hotel unit from 2020 to 2021 (103 euros):
[2]-[1]
429 -949 -750 561 476 -3,853 -4,538
5

[4] Percent variation
per hotel unit from 2020 to 2021
-14.4 47.8 81.1 -53.2 -51.5 288.0 183.6 -7.0

Table 7. | Pandemic impacts – simulation results
In this table, impacts are calculated as the difference between the real values and the Monte Carlos simulation estimates (impacts measured in 103 euros), and the percentage of these estimates (ratio between the impact and the real value). The precision control settings were activated in Crystal Ball and set to ensure the simulation trials would stop when the standard 95 percent confidence level was reached.

Trials Operating revenues Mean Standard deviation Impact (103 euros) Impact (percentage) Net total assets Mean Standard deviation Impact (103 euros)

1 50
8,007,468 450,331 9,090,638 113.5
23,885,532 1,343,295 -6,787,426

2 50
8,060,278 365,999 9,037,829 112.1

24,043,058 1,091,740 -6,944,952

3 50
7,998,740 376,435 9,099,366 113.8
23,859,498 1,122,870 -6,761,392

Simulation experiments

4

5

6

7

50

50

50

50

8,044,370 307,269 9,053,737 112.6

8,034,102 335,789 9,064,004 112.8

8,099,004 379,379 8,999,102 111.1

7,947,705 446,442 9,150,401 115.1

23,995,606 916,556
-6,897,500

23,964,978 1,001,628 -6,866,872

24,158,574 1,131,653 -7,060,468

23,707,264 1,331,696 -6,609,158

8 50
8,134,236 416,617 8,963,870 110.2
24,263,668 1,242,729 -7,165,562

9 50
8,052,962 381,428 9,045,144 112.3
24,021,237 1,137,765 -6,923,131

Mean of means 10
50

8,034,122 422,401 9,063,984 112.8

8,041,299 388,209 9,056,808 112.7

23,965,038 1,259,984 -6,866,932

23,986,445 1,157,991 -6,888,339

Impact (percentage) Net total debt Mean Standard deviation
Impact (103 euros) Impact (percentage) Net income Mean

Standard deviation Impact (103 euros) Impact (percentage) Operating cash flow Mean Standard deviation Impact (103 euros) Impact (percentage) Financial slack Mean Standard deviation Impact (103 euros) Impact (percentage) Debt capacity Mean Standard deviation Impact (103 euros)
Impact (percentage) Excess cash holdings Mean Standard deviation Impact (103 euros) Impact (percentage) Cash flow at risk CFaR (103 euros)

-28.4
7,863,357 1,134,950 9,234,749
117.4
879,005 48,800 -299,829 -34.1
1,332,897 26,720 44,543 3.3
8,329,951 240,764 -2,317,675
-27.8
17,307,578 956,880
-15,930,138 -92.0
271,133 15,248 -818,465 -301.9
1,289,972

-28.9
7,996,383 956,880 9,101,723 113.8
884,727 39,658 -305,551 -34.5
1,329,954 21,900 47,486 3.6
8,301,003 194,632 -2,288,727
-27.6
17,419,005 778,808 -
16,041,565 -92.1
272,921 12,393 -820,253 -300.6
1,294,724

-28.3
7,841,280 948,802 9,256,826 118.1
878,063 40,782 -298,887 -34.0
1,333,629 22,545 43,811 3.3
8,333,433 199,436 -2,321,156
-27.9
17,287,769 801,754 -
15,910,328 -92.0
270,837 12,746 -818,169 -302.1
1,296,050

-28.7
7,956,239 774,494 9,141,867 114.9
883,004 33,287 -303,828 -34.4
1,331,042 18,460 46,399 3.5
8,308,932 162,399 -2,296,655
-27.6
17,384,576 654,855 -
16,007,136 -92.1
272,382 10,404 -819,714 -301.0
1,299,094

-28.7
7,930,379 846,357 9,167,727 115.6
881,892 36,378 -302,716 -34.3
1,331,599 20,115 45,841 3.4
8,314,553 177,805 -2,302,277
-27.7
17,362,909 715,285 -
15,985,469 -92.1
272,035 11,370 -819,366 -301.2
1,299,968

-29.2
8,094,007 956,244 9,004,099 111.2
888,925 41,103 -309,749 -34.9
1,327,586 22,787 49,854 3.8
8,280,469 200,972 -2,268,192
-27.4
17,501,484 808,068 -
16,124,044 -92.1
274,232 12,846 -821,564 -299.6
1,293,503

-27.9
7,712,722 1,125,151 9,385,384
121.7
872,531 48,376 -293,355 -33.6
1,336,479 26,482 40,961 3.06%
8,361,543 238,491 -2,349,266
-28.1
17,180,219 948,816 -
15,802,779 -92.0
269,109 15,117 -816,441 -303.4
1,291,871

-29.5
8,182,854 1,050,063 8,915,252
109.0
892,742 45,147 -313,566 -35.1
1,325,352 24,946 52,088 3.9
8,262,287 238,491 -2,250,011
-27.2
17,577,051 886,185 -

16,199,611 -92.2
275,425 14,107 -822,757 -298.7
1,289,014

-28.8
7,977,961 961,319 9,120,145 114.3
883,933 41,330 -304,757 -34.5
1,330,352 22,676 47,088 3.5
8,305,212 203,389 -2,292,935
-27.6
17,403,774 811,029 -
16,026,334 -92.1
272,673 12,915 -820,005 -300.7
1,294,308

-28.7

-28.7

7,930,506 1,064,609 9,167,600
115.6

7,948,569 981,887 9,149,537 115.2

881,894 45,771 -302,718 -34.3

882,672 42,063 -303,495 -34.4

1,331,383 25,188 46,057 3.5

1,331,027 23,182 46,413 3.5

8,315,326 225,078 -2,303,050
-27.7

8,311,271 208,146 -2,298,994
-27.7

17,363,812 898,332
-15,986,372 -92.1

17,378,818 826,001
-16,001,378 -92.1

272,035 14,303 -819,367 -301.2

272,278 13,145 -819,610 -301.0

1,288,348

1,293,685

Table 8. | Robustness checks on the pandemic impacts (2020-2021)

This table presents: the real aggregate impact for the 2020-21 period [1]; the deterministic estimations for output variables (see [2]); the Monte Carlo (MC) estimations for output variables (see [3]); the aggregate impact for the 2020-21 period considering MC estimates in 103 euros [4] and percent [5]; the differences between the pandemic deterministic and MC impacts as a percent of real [6]; deviation of the two estimates as a percent of the deterministic estimates [7].

Output variables

[1] Real 2020-21
(103 euros)

[2] Deterministic estimates 2020-21
(103 euros)

Operating revenues

2 970 967

8 288 356

Net Total Assets

17 098 106

24 723 395

Net Total Debt

6 012 276

8 569 634

Net Income

- 547 332

910 443

Operating Cash Flow

579 176

1 317 470

Financial Slack

1 529 487

8 078 267

Debt capacity

8 357 450

17 801 258

Excess cash holdings

152 047

280 644

Sources: ORBIS and SABI databases; authors' estimations.

[3] MC estimates 2020-21 (103
euros) 8 041 299 23 986 445 7 948 569
882 672 1 331 027 8 180 446 17 378 818
272 278

[4] Real-MC estimates 2020-21 (103 euros) - 5 070 332 - 6 888

339 - 1 936 292 - 1 430 003 - 751 851 - 6 650 959 - 9 021 368 -
120 231

[5] Real-MC est. as percent of MC estimates
-63.1 -28.7 -24.4 -162.0 -56.5 -81.3 -51.9 -44.2

[6] percent
[3] – [1]
1.1 2.1 5.5 -1.9 -0.5 -0.2 1.1 1.7

[7] percent
[4] / [3]
-1.7 -6.9 -18.4 1.2 0.8 0.3 -2.2 -3.6

Appendix I – Sustainable growth model derivation

Following Van Horne (2002), Zantout (1990), and Higgins (1977), we derived a steady-state version of the sustainable growth rate model to estimate the 2020 and 2021 income statements, balance sheets, and cash flow statements for a sample of the Portuguese mainland hotel industry. The model was derived under the assumption that balances of balance sheet accounts are optimized in relation to the current level of sales and that depreciations were not an available source of funds because an application of the same amount in fixed assets is assumed to sustain their operational functionality.

Variable S
C&E / S RCV / S INV / S OCA / S FAS / S PAY / S OCL / S NIC
/ S
r D/E

Specification Operating revenue Cash & equivalents / Sales Receivables / Sales Inventory / Sales Other current assets / Sales Fixed assets / Sales Payables / Sales Other current liabilities / Sales Net income / Sales Net income - dividends Debt / Equity

where C&E denotes cash and equivalents; S, operating revenue; RCV, receivables; INV, inventory; OCA, other current assets; FAS, net fixed assets; PAY, payables; OCL, other current liabilities; NIC, net income; D, Debt; E, Equity; r, retention rate; g denotes sales sustainable growth rate.

C

& S

E

DS

+

RCV S

DS

+

INV S

DS

+

OCA S

DS

+

FAS S

DS

=

r

NIC S

(S

+

DS

)

+

ëéêr

NIC S

(S

+

DS

)ùûú

D E

+

PAY S

DS

+

OCL S

DS

C

& S

E

DS

+

RCV S

DS

+

INV S

DS

+

OCA S

DS

+

FAS S

DS

=

r

NIC S

(S

+

DS

) çæè1 +

D E

ö ÷ø

+

PAY S

DS

+

OCL S

DS

C

&E S

DS

+

RCV S

DS

+

INV S

DS

+

OCA DS S

+

FAS S

DS

=

r

NIC S

æèç1+

D E

ö ø÷

S

+

r

NIC S

æçè1 +

D E

ö ø÷

DS

+

PAY S

DS

+

OCL S

DS

C

&E S

DS

+

RCV S

DS

+

INV S

DS

+

OCA DS S

+

FAS S

DS

-

r

NIC S

æçè1+

D E

ö ø÷

DS

-

PAY S

DS

-

OCL S

DS

=

r

NIC S

æçè1+

D E

ö ÷ø

S

DS

é ëê

C

& S

E

+

RCV S

+

INV S

+ OCA S

+

FAS S

-

PAY S

- OCL S

-r

NIC S

èçæ1+

D öù E ÷øûú

=

r

NIC S

æèç1+

D E

ö ÷ø

S

g = DS =

r

NIC S

æèç1 +

D E

ö ÷ø

S

C

& S

E

+

RCV S

+

INV S

+

OCA S

+

FAS S

-

PAY S

-

OCL S

- ëêér

NIC S

çèæ1 +

D E

öù ÷øúû

20

Appendix II – Variable specification

Panel A | Income statement Variable
Operating revenue Sales Cost of sales Operating costs Depreciation EBIT Net interest expense P/L before tax Income taxes Net income Paid out dividends Retained earnings

Specification
Net Salest + Inventory variationt, Net Salest-1 x (1 + g) operating revenuest x (cost of sales2019/operating revenues2019) Labor Costst + Other Operating Costst Operating revenuest x (Depreciation2019/Operating revenues2019) Operating Revenue - Cost of Sales - Operating Costs - Depreciation Interest paidt + Incremental Financial Expenset – Incremental Financial Revenuet EBIT - Net interest expense P/L before tax x Income tax rate P/L before tax - Income taxes Net incomet x [Net income2019 – (Equity2019 - Equity2018)] / net income2019 Net incomet - Paid out dividendst

Panel B | Balance sheet Variable
Fixed assets Current assets
Inventory Receivables Other current assets Cash & equivalents TOTAL ASSETS Equity Liabilities Non-current liabilities Funding

needs Current liabilities TOTAL EQUITY + LIABILITIES

Specification
Fixed assetst / SalesI Inventoryt + Receivablest + Other Current Assetst + Cash & Equivalentst Inventoryt / Salest Receivablest / Salest Other Current Assetst / Salest Operating revenuest x Cash & equivalents2019/ Operating revenues2019
Equityt-1 + Retained Earningst
Total Assetst – Equityt - Non-current Liabilitiest - Current Liabilitiest Payablest + Other Current Liabilitiest

Panel C | Cash flow statement

Variable

Specification

Operating Cash Flow (gross) Δ Working capital Operating Cash Flow

Net Interest Expenset + Depreciation & Amortizationt Δ Receivablest + Δ Inventoriest - Δ Payablest Operating Cash Flow (gross)t - Δ Working capitalt

Panel D | Financial slack Variable
Debt Capacity
Excess Cash Holdings Liquidity Buffer Defense Interval
Financial Slack

Specification
(Earnings Before Interest & Taxest / Interest Coverage Ratiot) / (rF + Credit Risk Premium Cash & Equivalentst – Liquidity

Buffert Defense Intervalt x Daily Operating Expensest Current Assetst / Daily Operating Expensest Debt Capacityt – Non-Current Liabilitiest + Excess Cash Holdingst

21

End of report Thanks for using Viper
